# Novel Insight into the Therapeutic Targets for Spinal Degenerative Diseases Gained by a Post-Genome-Wide Association Study

**DOI:** 10.7150/ijms.127489

**Published:** 2026-05-18

**Authors:** Xuan Zhao, Qijun Wang, Chunjie Wang, Haixia Huang, Peng Wang, Wei Wang, Jie Lu, Shibao Lu

**Affiliations:** 1 Department of Orthopedics, Xuanwu Hospital, Capital Medical University, National Clinical Research Center for Geriatric Diseases, No.45 Changchun Street, Xicheng District, Beijing, 100053, China.; 2 Laboratory for Clinical Medicine, Capital Medical University, Beijing, China.; 3 Department of Radiology and Nuclear Medicine, Xuanwu Hospital, Capital Medical University, No. 45 Changchun Street, Xicheng District, Beijing, 100053, China.; 4 Department of Physiology and Pathophysiology, School of Basic Medical Sciences, Capital Medical University, Beijing, 100069, China.

**Keywords:** degenerative spinal diseases, Mendelian randomization, phenome-wide association analysis, druggable genes, single-cell RNA sequencing, molecular docking

## Abstract

Degenerative spinal diseases are a group of conditions that affect the structure and function of the spine, which can severely impact patient prognosis. Elucidating the druggable genes governing degenerative spinal diseases plays a pivotal role. GWAS data on degenerative spinal diseases, druggable genes, and eQTL data were acquired and Mendelian Randomization (MR) analysis was conducted. Colocalization analysis was performed and the side effects or additional indications of the identified drug targets was assessed using a phenome-wide association study. Finally, the differential expression of druggable genes in different cell subtypes of osteoporosis and intervertebral disc degeneration was analyzed, and molecular docking of actionable drugs for target genes was performed. In total, 31 druggable genes associated with osteoporosis and 22 druggable genes associated with spinal stenosis were identified. Four genes reached significant colocalization in blood for osteoporosis and eleven genes showed colocalization in spinal stenosis blood. Besides GPX1, KLRC2, and TCF19, the other 13 potential therapeutic targets for spinal degenerative diseases had no significant adverse effects. Furthermore, it was found that PAK4 and ESR1 were highly expressed in mesenchymal stem cells (MSCs) derived from bone marrow (BM-MSCs) in osteoporosis samples. SORCS2 expression was higher in nucleus pulposus cells (NPCs) in samples with severe than in samples with mild intervertebral disc degeneration. Lastly, drug analysis supported Fostamatinib might be potential therapeutics for osteoporosis and spinal stenosis by inhibiting PAK4 and PI4KB, respectively. In conclusion, our research suggested that PAK4, ESR1, and PI4KB might be promising targets for spinal degenerative diseases, and the effectiveness of Fostamatinib in these diseases warrants further investigation.

## 1. Introduction

Degenerative spinal diseases encompass a group of conditions affecting the spine and associated tissues, including osteoporosis, intervertebral disc degeneration, and spinal stenosis, etc.^1^, ^2^. These diseases often result in pain, functional impairment, and decreased quality of life, significantly impacting the health and well-being of patients^3^, ^4^. The pathogenesis is multifaceted, involving a complex interplay of genetic, environmental, and lifestyle factors^5^, but also is closely associated with metabolic microenvironment imbalance. This imbalance is often triggered by nutritional supply disorders or age-related oxidative stress, thereby inducing the homeostasis disturbance of chondrocytes, osteoblasts and nucleus pulposus^6^. Osteoporosis is primarily associated with an imbalance in bone tissue turnover and a reduction in bone mass^7^, while intervertebral disc degeneration and spinal stenosis are closely related to degenerative changes in intervertebral discs and vertebral bodies, as well as abnormalities in spinal canal structure^8^, ^9^. Currently, treatment mainly involves medication, physical therapy, and surgical intervention^10^, ^11^. Identifying the specific drug targets for these diseases and intervening at the molecular level in the early stage of the disease is an important strategy for treating these diseases. However, the drugs currently used for treating these diseases are mainly for alleviating symptoms, lacking specificity or having certain adverse reactions. Therefore, it is urgent to clarify the underlying mechanisms and develop effective treatment methods.

Druggable genes refer to genes that can serve as drug targets or be involved in drug development when treating specific diseases. These genes play crucial roles in the occurrence, progression, or treatment of diseases^12^. Drug therapy, as one of the important treatment modalities for degenerative bone diseases^13^, holds significant importance in regulating key genes. In recent years, an increasing number of studies have revealed druggable genes closely associated with diseases, including those related to orthopedics. Dieguez-Gonzalez and colleagues discovered that the druggable gene EDG2 is closely associated with the development of osteoarthritis^14^. Zhao et al. also reported that the druggable gene RIPK2 may serve as a therapeutic target for autoimmune diseases, including rheumatoid arthritis^15^. Additionally, Cao et al. identified potential drug targets for rheumatoid arthritis^16^. These studies all suggest that identifying druggable genes associated with orthopedic degenerative diseases is crucial for the treatment of these conditions. With the continued advancement of Mendelian randomization (MR) analysis, drug target MR has revealed an increasing number of drug targets that are causally related to diseases, providing new research directions for targeted therapy^16^, ^17^. Li et al., through MR analysis, indicated that bezafibrate and fenofibric acid may have potential roles in the prevention and treatment of osteoporosis^18^. Huang X et al., found that ARBs and thiazide diuretics play a crucial role in bone health based on MR analysis^19^. The above studies indicated that two-sample MR analysis plays a crucial role in uncovering drug targets for orthopedic diseases. However, the druggable gene map for degenerative spinal diseases is still incomplete. Even the latest genome-wide MR studies have only preliminarily screened out candidate genes, but have not yet clearly defined their core regulatory roles in the occurrence and development of the disease, and lack in-depth analysis of the gene functions and action pathways. Moreover, most existing studies are limited to a single gene, and fail to integrate the interaction network of “gene-cell-microenvironment”, making it difficult to identify the key druggable genes with global regulatory effects.

In this study, we employed a method combining drug target MR and colocalization to analyze specific drug targets for various spinal degenerative diseases. Additionally, we explored the differences in cell types involved in the onset and development of osteoporosis through single-cell analysis, as well as the variations of osteoporosis drug target genes among different cell types. This approach offers new insights and strategies for the treatment of spinal degenerative diseases.

## 2. Materials and Methods

### 2.1 Collection of GWAS data for spinal degenerative diseases

**Figure 1:**
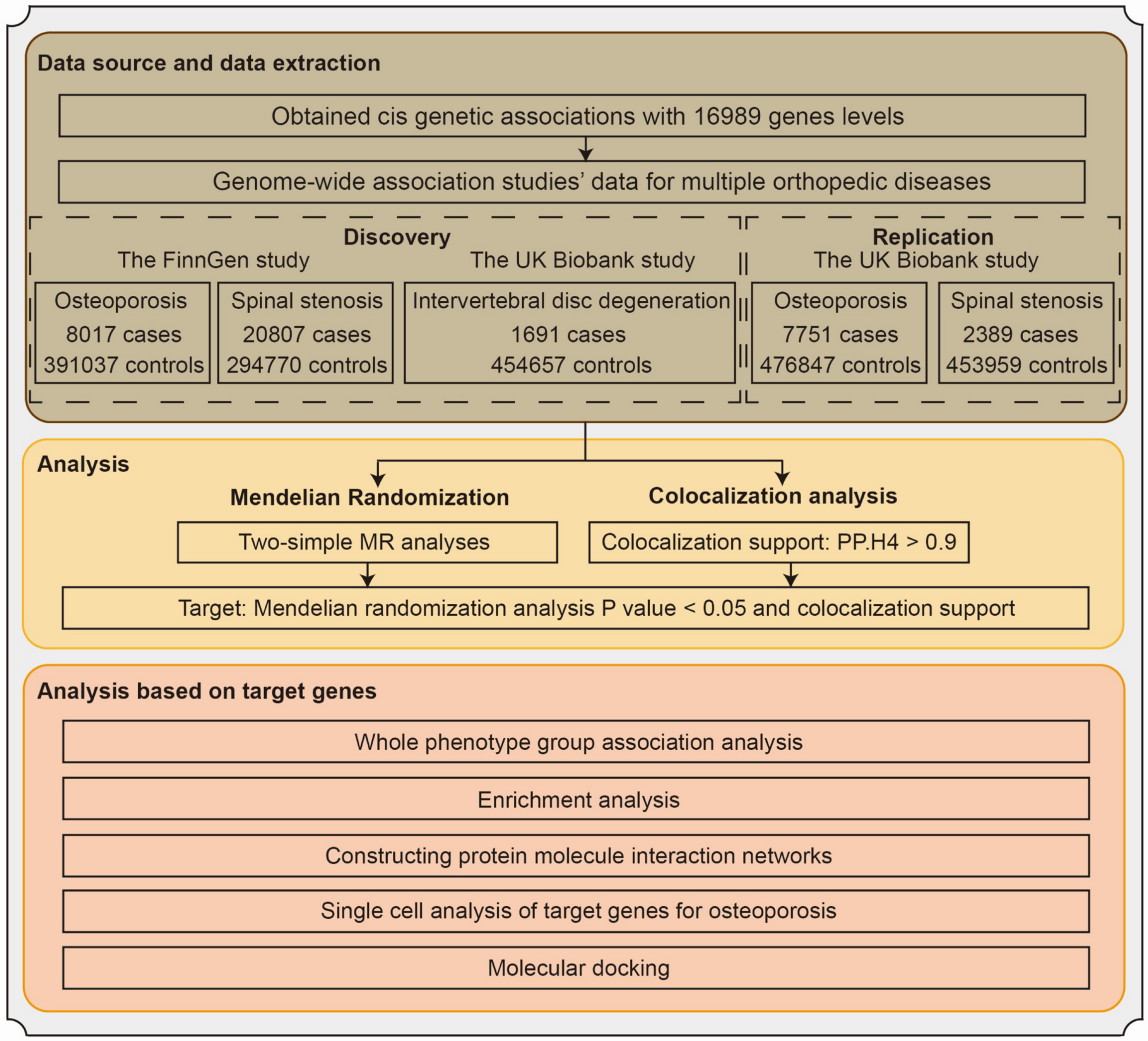
Overall study design based on Mendelian randomization (MR) and analysis related to key drug targets.

The purpose of this study is to elucidate the druggable genes governing degenerative spinal diseases, as seen in Fig. 1. A total of 21 different GWAS summary datasets of spinal degenerative diseases in European populations were obtained from databases such as the GWAS Catalog, IEU and Finngen, including osteoporosis, spinal stenosis, and disc degeneration, among others (Table 1).

### 2.2 Source of single-cell RNA sequencing (scRNA-seq) data

The scRNA-seq dataset was sourced from the Gene Expression Omnibus (GEO) database. The osteoporotic sample was included in the GSE147287 dataset, namely GSM4423510, while healthy donor bone marrow samples were from GSE120446 dataset. Six control samples were included in the GSE120446 dataset, namely GSM3396163, GSM3396165, GSM3396179, GSM3396180, GSM3396181 and GSM3396182. The data of intervertebral disc degeneration were sourced from the GSE244889 dataset, which included four mild intervertebral disc degeneration (GSM7831813, GSM7831814, GSM7831815, and GSM7831816 r) and three severe intervertebral disc degeneration (GSM7831817, GSM7831818, and GSM7831819), corresponding to the control and degenerated group respectively.

### 2.3 Acquisition of druggable genes and eQTL data

Druggable genes were retrieved from the DGIdb database, which provided comprehensive information on drug-gene interactions and drug-targetable genes. This retrieval included all genes classified under the "druggable" category within the DGIdb database. Then, a list of druggable genes were obtained from a review by Finan et al^20^. In addition, the eQTL dataset used in this study was obtained from eQTLGen, which provides transcriptome-wide eQTL data for 16,989 genes from 31,684 blood samples from healthy individuals of European ancestry.

### 2.4 MR analysis of genes and multiple spinal degenerative diseases

In our study, using the eQTL data of drug-target genes as exposure data and multiple spinal degenerative diseases as outcomes, MR analysis was implemented using the R package “TwoSampleMR”. The analysis strictly adhered to the MR reporting guidelines for observational studies (STROBE-MR)^21^. SNPs with FDR < 0.05 and within ± 100 kb of the transcription start site (TSS) of each gene were selected, and linkage disequilibrium analysis (r^2^ < 0.01, kb = 10,000) was performed. The PhenoScanner database was used to search for relevant SNPs using keywords such as Osteoporosis, Osteoporosis with pathological fracture, Drug-induced osteoporosis, Spinal stenosis, and Degeneration of intervertebral disc, and SNPs associated with these spinal diseases were removed from the analysis. The Wald ratio method was employed to compute the MR estimate for each SNP. When multiple SNPs were available, the weighted average of the ratio estimates was calculated, weighted by inverse variance weighted (IVW). Finally, sensitivity analyses were carried out to assess the robustness of the findings and address any heterogeneity and horizontal pleiotropy associated with the instruments. MR analysis relies on three key assumptions: (a) the association assumption, which requires instrumental variables (IVs) to be strongly linked to the exposure; (b) the exclusivity assumption, stating that IVs should not be directly connected to the outcome, meaning there is no horizontal pleiotropy effect between IVs and the outcome; and (c) the independence assumption, which dictates that IVs should not be associated with any potential confounding factors^22^.

### 2.5 Colocalization analysis of SNPs associated with the druggable gene eQTL and multiple spinal degenerative diseases

This study assigned the prior probability for SNPs associated with eQTL as P1, the prior probability for SNPs associated with different spinal diseases as P2, and the prior probability for SNPs simultaneously associated with eQTL and different spinal degenerative diseases as P12. When conducting tests using the R package coloc, default prior probabilities were used: P1 = 1×10^-4^, P2 = 1×10^-4^, P12 = 1×10^-5^, focusing on SNPs in the cis-eQTL region for analysis. Subsequently, posterior probabilities (PP) were utilized to quantify the credibility of all hypotheses, denoted as PPH0 - PPH4. Specifically, PPH0 represents the hypothesis where SNPs within the selected region show no significant correlation with either quantitative traits or the onset of spinal diseases. PPH1 refers to the hypothesis where SNPs within the selected loci are associated with quantitative traits but not with the risk of spinal disease onset. PPH2 corresponds to the hypothesis where SNPs within the selected loci are associated with the risk of spinal disease onset but not with quantitative traits. PPH3 represents the hypothesis where SNPs within the selected loci are associated with both the risk of spinal disease onset and quantitative traits, with these associations being independent of each other. Finally, PPH4 denotes the hypothesis where SNPs within the selected loci are associated with both the risk of spinal disease onset and quantitative traits, and these SNPs are shared between the two associations. Candidate drug target genes were defined as those identified through colocalization analysis of eQTL and spinal degenerative disease, showing evidence of shared colocalization (PPH4 > 0.9).

### 2.6 Phenome-wide MR analysis

The genome-wide MR analysis was conducted using druggable gene expression as the exposure and 783 traits (diseases) as outcomes. Subsequently, the same parameters were used to conduct an MR analysis using blood eQTLs of the druggable genes, further exploring the potential side effects of the identified drug-target genes.

### 2.7 Enrichment analysis

To further predict the potential molecular functions of druggable genes, enrichGO and enrichKEGG functions of the “clusterProfiler” package were used to perform Gene Ontology (GO) and Kyoto Encyclopedia of Genes and Genomes (KEGG) enrichment analyses, respectively.

### 2.8 Protein-protein interaction (PPI) network construction

To elucidate the genes that interact with key drug-target genes, the String database was used to input the selected drug-target genes, a protein interaction network was drawn, and the interaction with other genes was further analyzed.

### 2.9 Single cell analysis

Quality control and analysis on the scRNA-seq data were carried out using the R package “Seurat” (v4.1.1). After data standardized and normalization, highly variable genes in each sample were identified using the FindVariableFeatures function. Subsequently, we conducted linear dimensionality reduction using RunPCA based on the highly variable genes and the top 30 principal components (PCs). The R package “harmony” was utilized to remove batch effects between samples. Following the integration and dimensionality reduction of the samples, preliminary clustering of the cells was performed using the FindClusters function.

### 2.10 Molecular docking

The protein structures of PAK4, ESR1, and PI4KB were obtained from the PDB database, while the structural information of small molecule drugs was obtained from the ZINC database. Subsequently, molecular docking was performed using AutoDock Vina, and protein-compound binding figures were generated using PyMOL.

### 2.11 Statistical analysis

Statistical analysis in this study was conducted using R version 4.2.1. The Wilcoxon test was applied to assess the differences between two groups of samples, and a p-value of less than 0.05 was considered statistically significant.

## 3. Results

### 3.1 The causal effect of druggable genes on spinal degenerative diseases

First, by integrating 3,952 potentially drug-resistant genes from the DGIdb database with 4,479 drug-resistant genes reported in the literature, a comprehensive set of 5,859 unique drug-resistant genes was compiled (Table S1). The eQTL data of potentially druggable genes were intersected with spinal degenerative diseases-related SNPs retrieved from the PhenoScanner database. SNPs associated with osteoporosis (rs79751840, rs10005067, and rs2598124) and spinal stenosis (rs17478394 and rs112874046) were excluded from the eQTL data. The remaining druggable genes were intersected with plasma cis eQTL data from the eQTLGene database, resulting in 3,627 genes for MR analysis. The eQTL and MR analysis results for 3,627 drug-targetable genes and 21 sets of multiple spinal disease outcomes were presented in Table S2 and Table S3. It could be observed that, except for GCST90080487 and GCST90080489, the remaining 19 sets of outcomes exhibited significantly causal associations (p-value < 0.05) with the corresponding phenotypes. An MR analysis was then conducted on the causally related druggable genes associated with spinal degenerative diseases. The results were presented in Table S4 and Figure S1 (forest plot), and these genes were considered potential drug target candidates.

**Figure 2:**
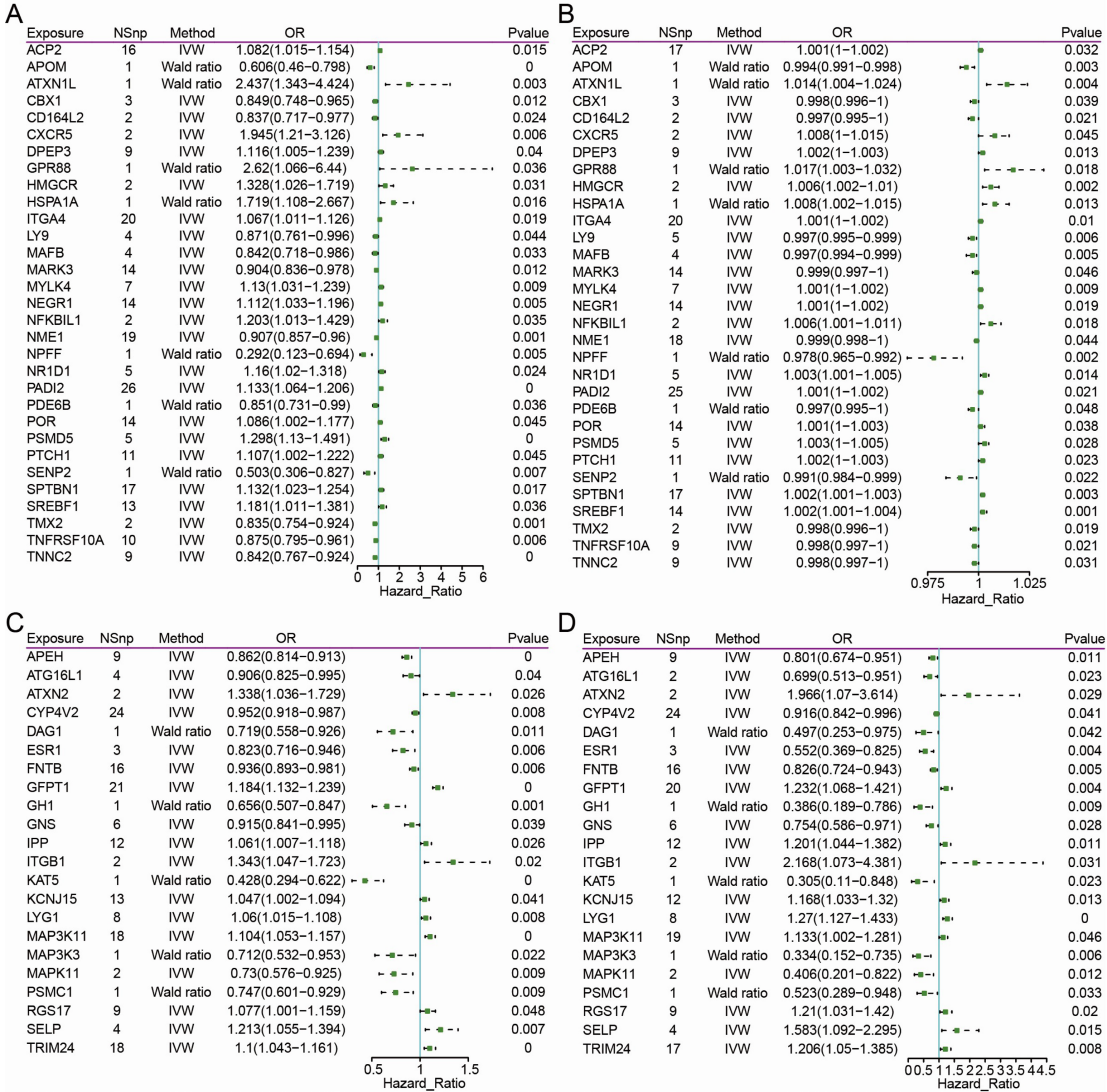
Forest plots showing the potential druggable genes associated with osteoporosis and spinal stenosis identified through MR analysis (osteoporosis: A: finngen_R10_M13_OSTEOPOROSIS; B: GCST90038656) (spinal stenosis: C: finngen_R10_M13_SPINSTENOSIS; D: GCST90044547).

In the subsequent analysis, osteoporosis and spinal stenosis were the primary focuses, with 14 and 4 outcome datasets, respectively. Significant causal relationships with drug targets were identified, and these potential drug targets were subsequently validated. The highest number of successfully validated genes in osteoporosis was observed in the finngen_R10_M13_OSTEOPOROSIS and GCST90038656 datasets, with a total of 31 drug target genes (OR greater than 1 or OR less than 1), as shown in Fig. 2A and B. Furthermore, in spinal stenosis, the most successful gene validation was achieved in the Finngen_R10_M13_SPINSTENOSIS and GCST90044547 datasets, with 22 drug target genes, as shown in Fig. 2C and D.

### 3.2 Colocalization analysis of potential druggable genes and multiple spinal degenerative diseases

To further determine the probability of sharing causal genetic variation with different spinal degenerative diseases and SNPs associated with eQTLs, colocalization analysis was conducted, which showed that four genes were significantly co-localized with osteoporosis in the blood, namely PAK4, KLRC2, ESR1, and TAS1R3; 11 genes were significantly co-localized with spinal stenosis in the blood, namely C9orf72, MEG3, TCF19, DLK1, IDH2, KDM6B, GPX1, TET2, PI4KB, CCL4, and PLA2G2C; and SORCS2 was significantly co-localized with degenerative disc disease in the blood (Table 2). Additionally, Table S5 showed the colocalization results of all potential drug targets and their corresponding MR analysis outcomes. The results of the colocalization analysis demonstrated that specific genes were significantly co-localized with various spinal degenerative diseases in the blood, highlighting potential genetic variations shared across these conditions.

### 3.3 Phenome-wide association study of druggable genes

**Figure 3:**
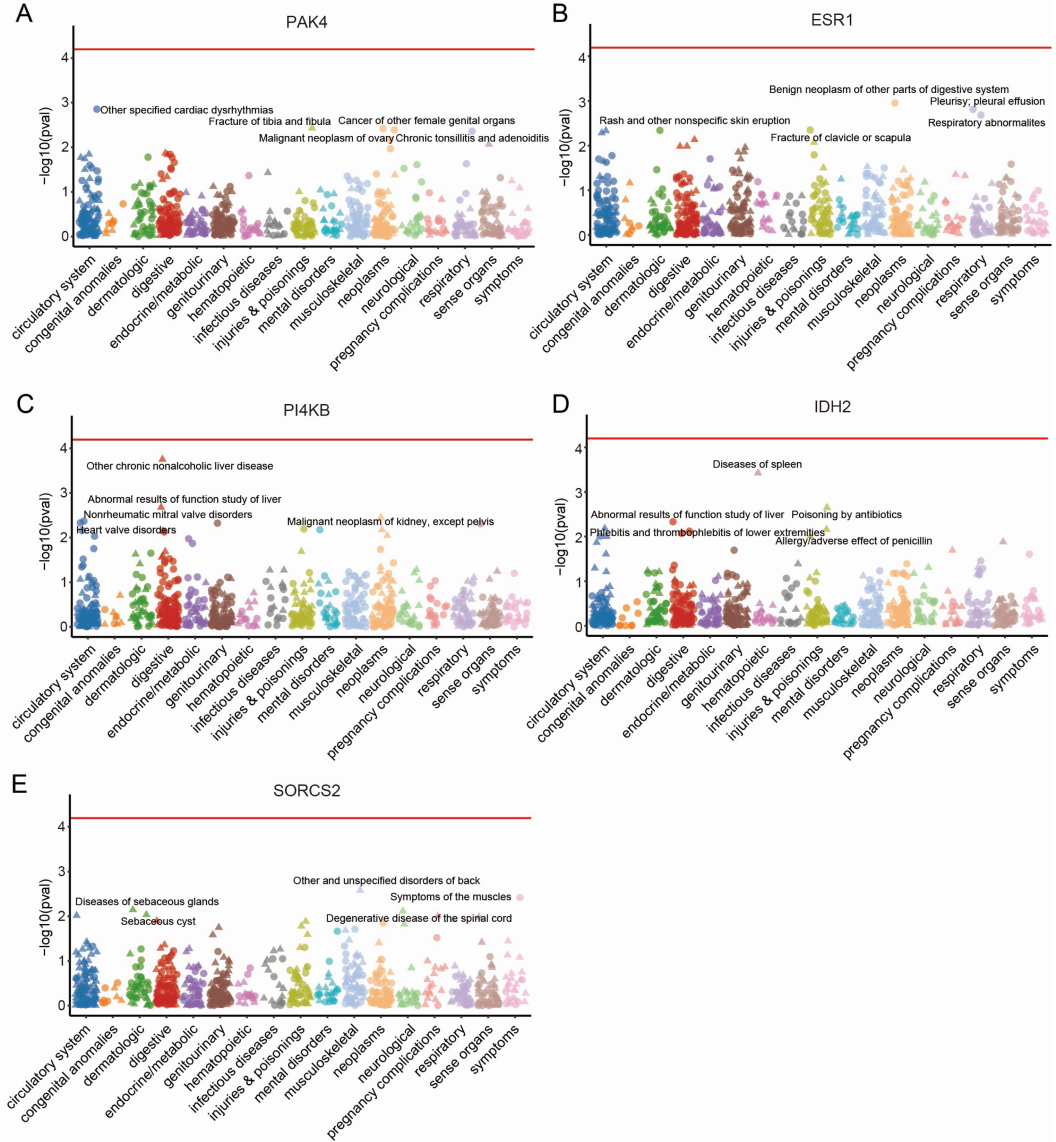
The full phenotypic MR analysis of the PAK4 (A), ESR1 (B), PI4KB (C), IDH2 (D), SORCS2 (E) genes. The circle represents the risk factor, while the triangle represents the protective factor.

Studies have suggested that most drugs exert their effects through the circulatory system^23^. Therefore, a phenome-wide association study was conducted to further evaluate whether the expression of 16 genes related to different spinal degenerative diseases in the bloodstream has beneficial or harmful effects on other indications. A more extensive MR screening was conducted for 783 diseases or traits, with the results presented in Table S6. The phenome-wide association analysis focused on the five druggable genes identified in osteoporosis, spinal stenosis and intervertebral disc degeneration. Causal effects were considered statistically significant when the p-value after Bonferroni correction was less than 6.386*10^-5, corresponding to -log10(p) > 4.194792 in genome-wide MR analysis. As shown in Fig. 3A-E, PAK4, ESR1, IDH2, PI4KB, and SORCS2 were not associated with 783 diseases and traits, indicating that drugs targeting these genes were unlikely to have potential adverse effects. Besides, an increase in the level of GPX1 in the blood may elevate the risk of essential hypertension and hypertension (Fig. S2 and Table S7). Higher blood KLRC2 levels have been found to be associated with a reduced risk of diseases of sebaceous glands, sebaceous cyst, degenerative disease of celiac disease, and intestinal malabsorption (non-celiac). Higher blood TCF19 levels may increase the risk of hypothyroidism NOS and hypothyroidism. In addition, the phenome-wide MR analysis for other drug target genes revealed that no associations were found between C9ORF72, CCL4, DLK1, KDM6B, KLRC2, MEG3, TAS1R3 and TET2 with 783 diseases and traits (Fig. S2 and Table S7).

### 3.4 Enrichment analysis elucidated the potential mechanism of action of druggable genes

**Figure 4:**
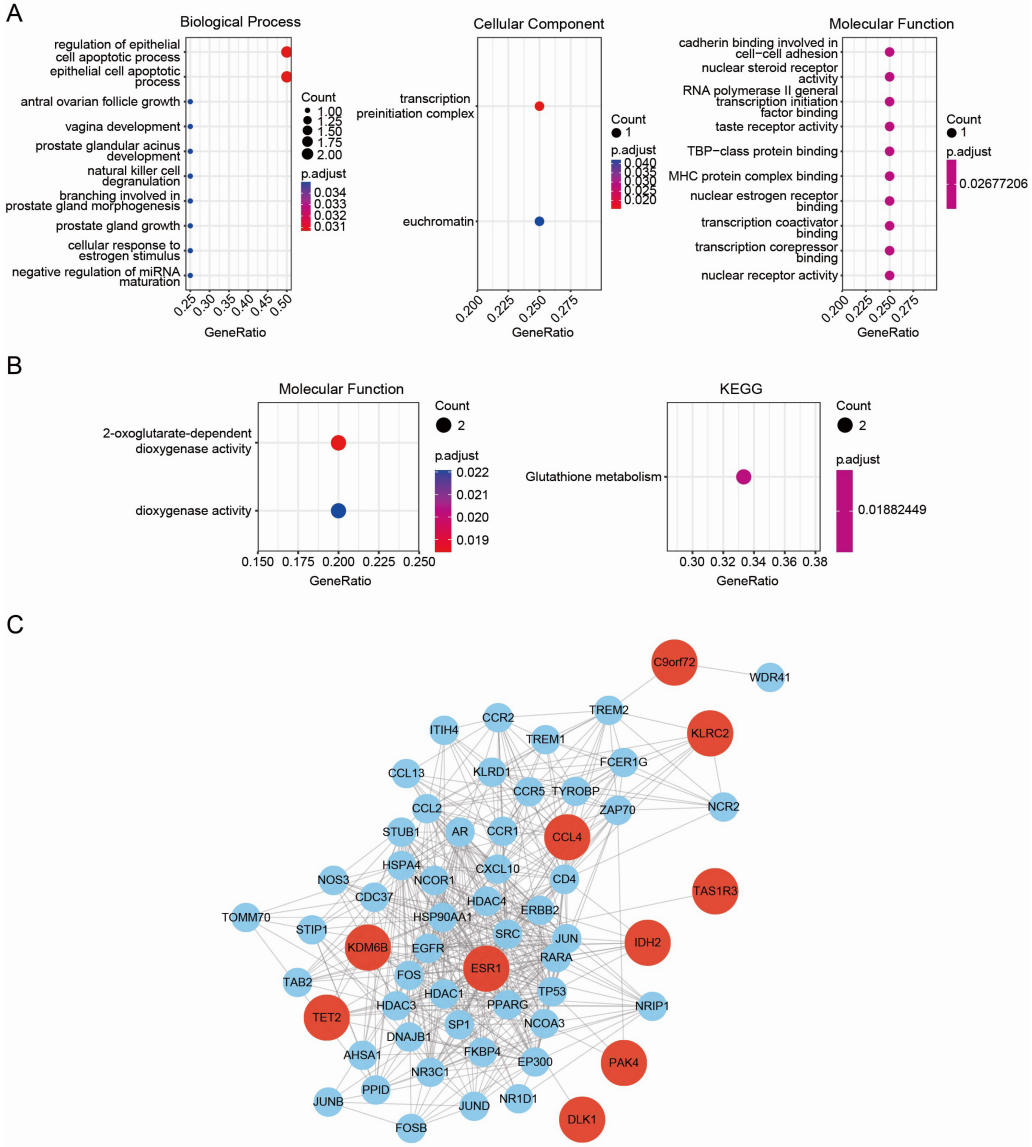
(A-B) Enrichment analysis result of osteoporosis and spinal stenosis related druggable genes. (C) Protein molecular interaction network of druggable genes.

To further elucidate the key signaling pathways of drug target genes in osteoporosis and spinal stenosis, we conducted GO and KEGG enrichment analyses of the drug target genes for these diseases, respectively (Fig. 4A and B). The drug target genes for osteoporosis were enriched in relevant biological processes in the GO database pathway, including regulation of epithelial cell apoptotic process, epithelial cell apoptotic process, etc. The cellular components were transcription preinitiation complex and euchromatin, and the molecular functions were cadherin binding involved in cell-cell adhesion, nuclear steroid receptor activity, etc. The drug target genes of spinal stenosis were only enriched in related molecular functions in the GO database pathway, including 2-oxoglutarate-dependent dioxygenase activity and dioxygenase activity, and were enriched in Glutathione metabolism in the KEGG database. Glutathione reductase (GR) is a core regulatory enzyme in the glutathione metabolic network. Studies^24^ have shown that high levels of GR in serum are closely related to the severity of intervertebral disc degeneration, the postoperative spinal fusion rate, and the relief effect of degeneration in patients with lumbar spinal stenosis accompanied by lumbar intervertebral disc protrusion. This association may essentially stem from the regulatory role of GR on the oxidative stress of intervertebral disc tissues, further suggesting that this target gene has significant potential value in degenerative spinal diseases.

### 3.5 PPI network revealed genes that interact with druggable genes

The druggable genes may interact with other genes, thereby influencing the development of degenerative spinal diseases. Therefore, a PPI network involving druggable genes was constructed, as shown in Fig. 4C. The graph revealed that interactions between ESR1 and genes such as EGFR, FOS, RARA, and SP1, while PAK4 interacted with genes such as NCOA3, TP53, and ZAP70. The above results indicated that identifying genes that interact with druggable genes is crucial for further elucidating the mechanisms by which key druggable genes regulate skeletal degenerative diseases.

### 3.6 scRNA-seq analysis revealed differential expression for key druggable genes among different cell types of osteoporosis

**Figure 5:**
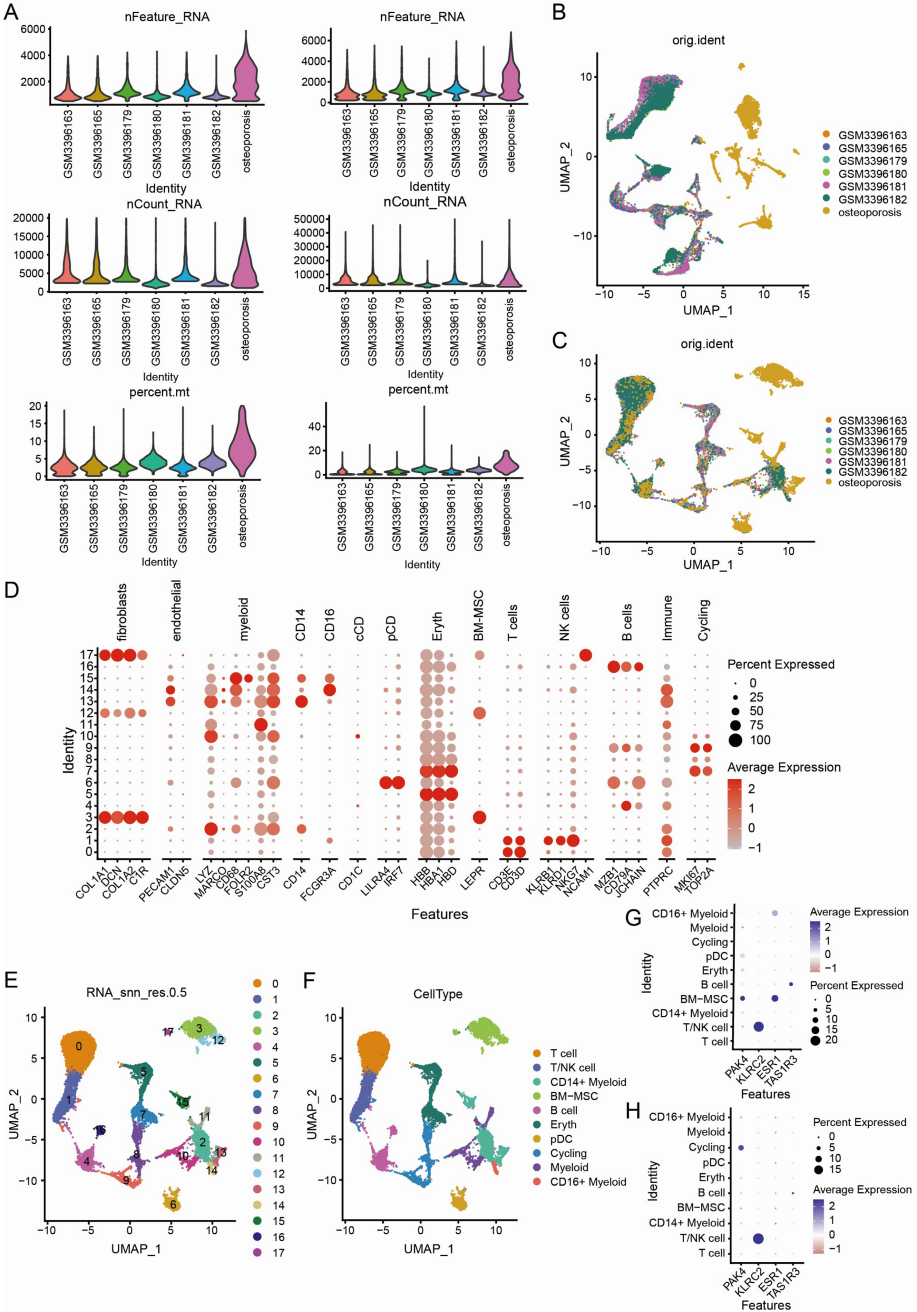
Single cell data of osteoporosis. (A) Comparison of various data before and after quality control; (B) Distribution of each sample before batch correction; (C) Distribution of each sample after batch correction; (D) The expression distribution of marker gene in each cluster; (E) Clustering result of single cell data sets; (F) Cell annotation result; (G) The expression distribution of druggable genes in each cell of osteoporosis sample; (H) The expression distribution of druggable genes in each cell of healthy bone marrow samples.

Quality control was first performed on the data, as shown in Fig. 5A, resulting in a total of 18,282 cells. Next, 2,000 highly variable genes were identified for principal component analysis (PCA) analysis, followed by batch correction (Fig. 5B and C). The cells were then annotated and clustered, revealing a total of 18 distinct cell clusters (Fig. 5D and E). The dataset included 18,282 cells, with the following distributions: T cells (4,973), T/natural killer (NK) cells (2,120), cycling cells (1,309), CD14^+^ myeloid cells (2,193), mesenchymal stem cells (MSCs) derived from bone marrow (BM-MSCs) (2,174), B cells (1,635), erythroid cells (1,750), plasmacytoid dendritic cells (pDC) (908), myeloid cells (963), and CD16^+^ myeloid cells (257) (Fig. 5F).

Afterwards, the expression distribution of identified osteoporosis-related druggable genes across different cell types in the single-cell dataset was analyzed, as shown in Fig. 5G and H. It was observed that in the osteoporosis samples, PAK4 and ESR1 were highly expressed in BM-MSCs, while KLRC2 was highly expressed in T/NK cells. In the healthy control samples, PAK4 was predominantly expressed in cycling cells, and KLRC2 was highly expressed in T/NK cells. These results indicated that these key druggable genes play a regulatory role in different cellular subtypes involved in osteoporosis.

### 3.7 scRNA-seq analysis related to disc degeneration revealed differential expression of SORCS2

**Figure 6:**
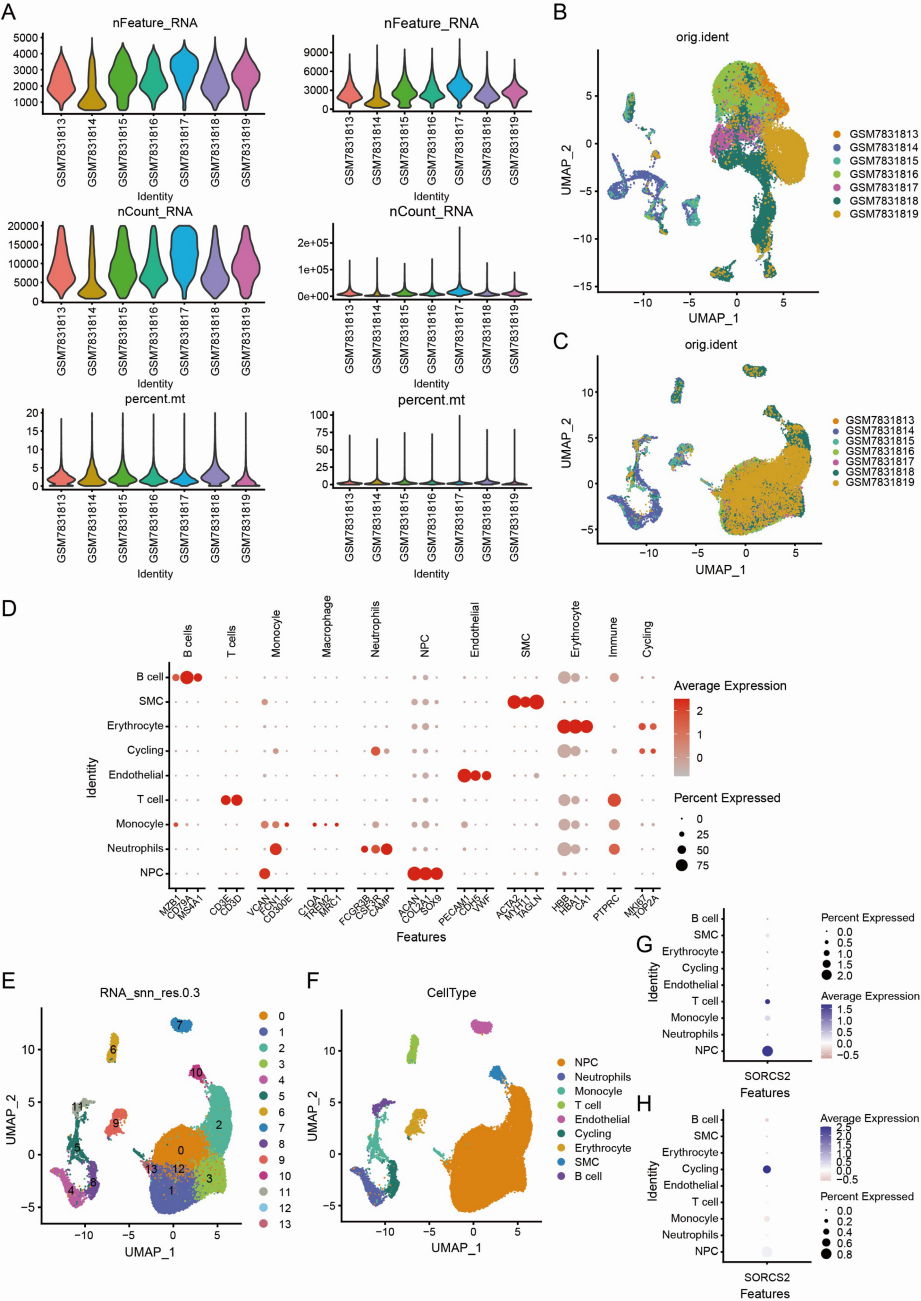
Single cell data of intervertebral disc degeneration. (A) Comparison of various data before and after quality control; (B) Distribution of each sample before batch correction; (C) Distribution of each sample after batch correction; (D) The expression distribution of marker gene in each cluster; (E) Clustering result of single cell data sets; (F) Cell annotation result; (G) The expression distribution of druggable genes in each cell of severe intervertebral disc degeneration sample; (H) The expression distribution of druggable genes in each cell of mild intervertebral disc degeneration.

Quality control was performed on the dataset for disc degeneration (Fig. 6A), resulting in a total of 44,299 cells. Harmony was then applied for batch correction (Fig. 6B and C). Cell annotation and clustering identified 18 cell clusters (Fig. 6D and E), comprising a total of 44,299 cells (in parentheses is the number of cells): nucleus pulposus cells (NPCs) (35,434), neutrophil (1,949), monocyte (1,270), T cell (1,180), endothelial (1,078), cycling (1,050), erythrocyte (999), smooth muscle cell (SMC) (688), and B cell (651) (Fig. 6F). Finally, the expression distribution of SORCS2 across different cell types in patients with severe and mild degenerative disc disease was analyzed (Fig. 6G and H). It was found that SORCS2 was highly expressed in NPCs in severe degenerative disc disease samples, whereas it was predominantly expressed in cycling cells in mild degenerative disc disease samples. These findings indicated that the regulation of SORCS2 in disc degeneration exhibited cellular heterogeneity.

### 3.8 Identification of operable drugs for target genes and molecular docking

**Figure 7:**
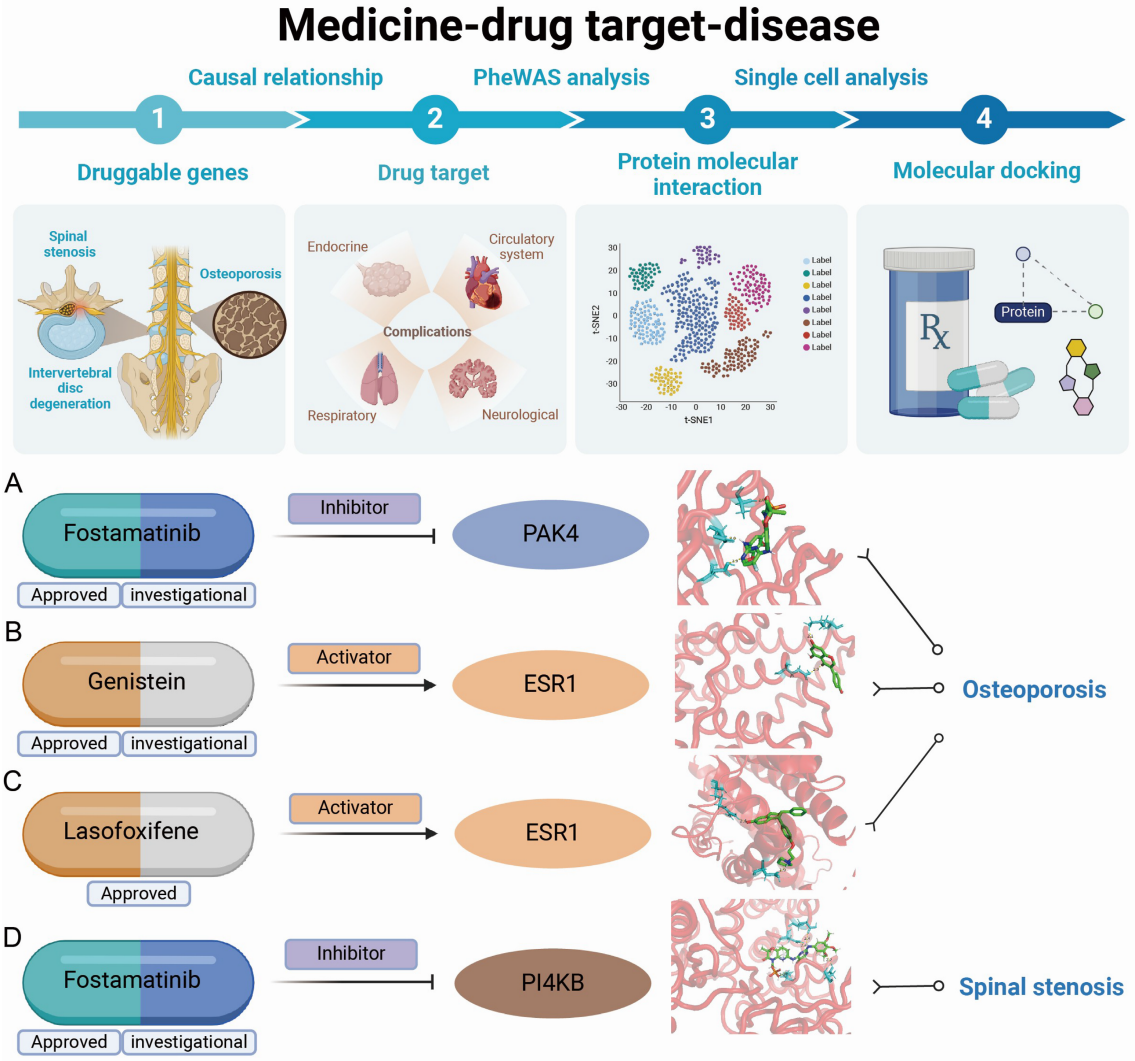
Interactions and molecular docking results between identified prospective drug targets and the present approved or investigational drugs. (A) PAK4 and Fostamatinib; (B) ESR1 and Genistein; (C) ESR1 with Lasofoxifene; (D) PI4KB with Fostamatinib.

The pre-clinical or clinical development activities of 16 candidate drug genes for three spinal degenerative diseases through Drugbank and DGIdb databases (Table S8). PAK4 and PI4KB were identified as risk factors for osteoporosis and spinal stenosis, respectively (Table S4). The current drug related to these two genes, Fostamatinib may serve as a potential therapeutic for osteoporosis and spinal stenosis by inhibiting PAK4 and PI4KB, respectively. ESR1, identified as a protective factor for osteoporosis (Table S4), has shown partial activation effect with Genistein and Lasofoxifene, which have been applied to some extent in the clinical treatment of osteoporosis. To further analyze the binding interactions between key drug-target genes and drugs, molecular docking was performed (Fig. 7A-D and Table 3). The results indicate that these key druggable genes can stably bind to their corresponding drugs. However, drugs targeting other candidate genes, such as IDH2 or SORCS2, may not be ideal, as they exhibit opposing effects on the three spinal degenerative diseases or have not been shown any activating or inhibiting effects on these genes.

## 4. Discussion

Spinal degenerative diseases significantly impact patients' quality of life, posing numerous challenges in treatment^25^. Druggable genes have played a crucial role in treating spinal degenerative diseases. Understanding and studying druggable genes associated with these conditions can aid in developing novel therapeutic strategies and medications targeting these diseases. In this study, four potential drug target genes associated with osteoporosis, eleven druggable genes associated with spinal stenosis, and one druggable gene closely related to intervertebral disc degeneration were identified using a two-sample MR analysis. Subsequently, based on single-cell analysis, it was found that in osteoporosis samples, PAK4 and ESR1 were highly expressed in BM-MSCs, while KLRC2 was highly expressed in T/NK cells. In healthy control samples, PAK was highly expressed in cycling cells, and KLRC2 was highly expressed in T/NK cells. Furthermore, it was discovered that in severe intervertebral disc degeneration samples, SORCS2 was highly expressed in NPCs, while in mild intervertebral disc degeneration samples, SORCS2 was highly expressed in cycling cells. Our research findings hold promise for providing theoretical foundations and practical guidance for the development of specific and effective drugs.

PAK4 (P21-activated kinase 4) is a member of the serine/threonine kinase family, and it is involved in cell signaling, cellular morphology, and cell motility^26^, ^27^. The research has found that PAK4 is closely associated with the development of various diseases^28^, including orthopedic degenerative diseases. Studies have shown that PAK4 regulates cell migration and podosome formation^29^, ^30^. Choi SW et al. also found a significant correlation between PAK4 and osteoclast differentiation, and osteoclast differentiation can be significantly inhibited by the PAK4 inhibitor PF-3758309^31^. Existing studies have shown that PAK4 enhances the survival, proliferation, and differentiation of osteoblasts by upregulating the expression of cyclin D1^32^. Based on our findings, we hypothesize that in osteoporosis, the elevated expression of PAK4 in BM-MSCs may promote adipogenesis, thereby reducing bone mass and contributing to the progression of osteoporosis^33^. This suggests that PAK4 could serve as a potential therapeutic target for osteoporosis and offers a novel strategy for improving the function of BM-MSCs.

The ESR1 gene encodes estrogen receptor alpha (ERα), a crucial nuclear receptor belonging to the nuclear hormone receptor superfamily. This receptor mediates the actions of estrogen within cells and is involved in regulating numerous physiological processes, including reproduction, bone health, cardiovascular health, and more^34^. Krela-Kaźmierczak et al. found that mutations in ESR1 can predict osteoporosis in female Crohn's disease patients^35^. Additionally, research has indicated an association between the ESR1 polymorphism rs9340799 and postmenopausal osteoporosis^36^. Furthermore, estrogen enhances the binding of ESR1 to Keap1, inhibiting the degradation of Nrf2. This activation of Nrf2-mediated antioxidant signaling promotes osteoblast activity and bone formation, helping to prevent osteoporosis^37^. Therefore, targeting the ESR1-Keap1-Nrf2 axis may have a potential therapeutic effect against osteoporosis, providing a promising clinical target for the treatment of menopause-related osteoporosis. This mechanism highlights its significant clinical value.

The protein encoded by the KLRC2 gene is a member of the human leukocyte antigen (HLA) family, also known as NKG2C. This protein serves as a ligand that interacts with the CD94 protein on the surface of NK cells, forming an immune complex, and plays a crucial role in immune responses, particularly in the activation and regulation of NK cells^38^, ^39^. Although no direct relationship between KLRC2 and osteoporosis has been reported in the literature, Aviles-Padilla et al. found that NKG2C^+^ NK cells exhibit unique functions after bone marrow transplantation^40^. Furthermore, NK cells, particularly NKT-like cells, can produce IL-4, a Th2 cytokine secreted by helper T cells, which effectively inhibits the differentiation and proliferation of osteoclasts^41^. NKG2C, by regulating NK cell function, especially through the secretion of IL-4, may indirectly influence osteoclast activity, thus playing a role in immune regulation of osteoporosis. Given that IL-4 can suppress osteoclast differentiation and proliferation, NKG2C holds potential as a target for further exploration in regulating bone resorption, offering a novel clinical target for immune-based therapies for osteoporosis.

The PI4KB gene encodes phosphatidylinositol 4-kinase beta (PI4KB), an essential enzyme in cellular membrane lipid metabolism. It plays crucial roles in cellular membrane assembly, cell polarity, signal transduction, and cell cycle regulation^42^. Mutations or dysregulation of the PI4KB gene have been associated with various diseases, including certain cancers, neurological disorders, and metabolic diseases^43^, ^44^. The SORCS2 gene encodes the SORCS2 protein, which belongs to the SORCS family of proteins and is a transmembrane receptor protein expressed widely in the nervous system. It is also associated with certain neurological disorders, such as Alzheimer's disease^45^, ^46^. There is currently no literature reporting the relationship between PI4KB and spinal stenosis, or between SORCS2 and intervertebral disc degeneration. Our study is the first to identify the crucial druggable gene PI4KB in spinal stenosis and the important druggable gene SORCS2 in intervertebral disc degeneration. These findings have significant implications for the treatment of both spinal stenosis and intervertebral disc degeneration. Additionally, we discovered that druggable genes associated with spinal stenosis are enriched in the critical Glutathione metabolism signaling pathway. Research by Fujita H and others has shown that glutathione can accelerate osteoclast differentiation and inflammatory bone destruction^47^. Researchers studying spinal stenosis have also found a close correlation between elevated glutathione reductase levels and the alleviation of the severity of spinal stenosis^24^. We speculate that Glutathione metabolism may play a role in spinal stenosis, but further evidence is needed to confirm this hypothesis.

Based on the drug prediction, we found that Fostamatinib has the potential to be a therapeutic drug for osteoporosis and spinal stenosis. Its core mechanism of action is to specifically target and inhibit PAK4 and PI4KB. As an oral prodrug of the spleen tyrosine kinase (Syk) inhibitor R-406, Fostamatinib blocks Fc/B cell receptor signaling in inflammatory cells, reduces TNFα/IL-1 release, exhibits anti-inflammatory and anti-bone resorption effects in rheumatoid arthritis (RA)-related preclinical/phase I/II studies, and boasts good oral bioavailability, tolerability, and gastrointestinal-predominant common adverse reactions^48^. Additionally, studies have confirmed that Fostamatinib has positive therapeutic effects on temporomandibular joint osteoarthritis *in vitro* and *in vivo* experiments, and its mechanism may be related to regulating the inflammatory response, ECM degradation, and abnormal cell apoptosis/autophagy of chondrocytes through the MAPK/NF-κB and AKT/mTOR pathways, thereby maintaining cartilage homeostasis^49^. Li H^50^ team identified Fostamatinib as a potential therapeutic drug for osteoporosis through the construction of a drug-target gene network, which is highly consistent with the conclusion of this study. In summary, existing studies provide multi-dimensional evidence for Fostamatinib's potential in treating osteoporosis, spinal stenosis, and other bone-related diseases, which supports the predictions of this study. However, further targeted cell and animal experiments as well as clinical research are required to confirm its specific mechanisms of action, optimal dosing regimen, and clinical efficacy, laying a solid foundation for robust clinical translation.

In summary, our study revealed the relationships of key druggable genes PAK4, KLRC2, and ESR1 with osteoporosis, PI4KB with spinal stenosis, and the crucial role of SORCS2 in intervertebral disc degeneration. These genes may serve as important targets for drug development in spinal degenerative diseases. Besides, this study, through a systematic Phenome-wide analysis, verified the safety of PAK4, ESR1, PI4KB and SORCS2 at the whole phenotypic level. This not only provides a clear direction for drug development, but also offers a treatment option with multiple benefits and extremely low side effects for clinical use.

However, this study has the following limitations: Firstly, the research data mainly come from the European population, which limits the generalization of the conclusions to other racial groups; Secondly, the potential molecular mechanisms by which the core drug target genes are involved in the occurrence and development of diseases lack functional validation and still require further exploration; Thirdly, the clinical relevance and drug transformation evidence are insufficient. The identified druggable genes need to be further verified in real-world patient populations, and the functional significance of these genetic associations in treatment research needs to be evaluated. These limitations have pointed out clear directions for future research, including expanding population diversity, deepening mechanism validation, and strengthening clinical transformation research.

## Supplementary Material

Supplementary figures and tables.

## Figures and Tables

**Table 1:** Detailed information on data of various orthopedic diseases

Accession	Trait	Source	Population	Sample size	nSNP	ncase	ncontrol
finngen_R10_M13_OSTEOPOROSIS	Osteoporosis	Finngen	EUR	399054	21306139	8017	391037
finngen_R10_OSTEOPOROSIS_FRACTURE_FG	Osteoporosis with pathological fracture (FG)	Finngen	EUR	313032	21303817	1822	311210
finngen_R10_OSTPOPATFRCTURE_POSTEMENO	Postmenopausal osteoporosis with pathological fracture	Finngen	EUR	230087	21298901	1486	228601
finngen_R10_OSTPOPATFRACTURE	Drug-induced osteoporosis with pathological fracture	Finngen	EUR	410747	21306323	388	410359
finngen_R10_DRUGADVERS_OSTEOPO	Drug-induced osteoporosis	Finngen	EUR	412181	21306330	307	411874
ukb-b-12141	Non-cancer illness code, self-reported: osteoporosis	IEU	EUR	462933	9851867	7547	455386
ukb-a-87	Non-cancer illness code self-reported: osteoporosis	IEU	EUR	337159	10894596	5266	331893
GCST90086118	Osteoporosis	GWAS_Catalog	EUR	56637	15066946	5399	51238
GCST90086119	Osteoporosis	GWAS_Catalog	EUR	56637	12116316	5399	51238
GCST90086120	Osteoporosis	GWAS_Catalog	EUR	56637	15103307	5399	51238
GCST90086121	Osteoporosis	GWAS_Catalog	EUR	56637	11419007	5399	51238
GCST90086122	Osteoporosis	GWAS_Catalog	EUR	56637	15544346	5399	51238
GCST90044600	Osteoporosis, not otherwise specified (PheCode 743.11)	GWAS_Catalog	EUR	456348	11842647	991	455357
GCST90038656	Osteoporosis	GWAS_Catalog	EUR	484598	9886868	7751	476847
ukb-d-M13_SPINSTENOSIS	Spinal stenosis	IEU	EUR	361194	10372237	1910	359284
GCST90044547	Spinal stenosis (PheCode 720)	GWAS_Catalog	EUR	456348	11842647	2389	453959
GCST90044553	Degeneration of intervertebral disc (PheCode 722.6)	GWAS_Catalog	EUR	456348	11842647	1691	454657
GCST90080487	ICD10 M48.02: Spinal stenosis, cervical region	GWAS_Catalog	EUR	387930	510688	607	387323
GCST90080488	ICD10 M48.06: Spinal stenosis, lumbar region	GWAS_Catalog	EUR	387930	510688	3396	384534
GCST90080489	ICD10 M48.0: Spinal stenosis	GWAS_Catalog	EUR	387914	510667	5125	382789
finngen_R10_M13_SPINSTENOSIS	Spinal stenosis	Finngen	EUR	315577	21303910	20807	294770

**Table 2:** Colocalization analysis results

nSNPs	PP.H0.abf	PP.H1.abf	PP.H2.abf	PP.H3.abf	PP.H4.abf	Gene	AccessionID	Trait
4230	2.22E-78	0.001363519	2.37E-77	0.013546232	0.985090249	TAS1R3	finngen_R10_M13_OSTEOPOROSIS	Osteoporosis
6655	3.22E-42	0.005329709	2.32E-41	0.037413261	0.957257031	ESR1	GCST90038656	Osteoporosis
3917	0	0.007508043	0	0.040406297	0.95208566	KLRC2	GCST90086119	Osteoporosis
5800	2.23E-144	0.053359959	1.97E-144	0.046323518	0.900316524	PAK4	GCST90086119	Osteoporosis
6890	0	0.017295132	0	0.019473475	0.963231393	C9orf72	finngen_R10_M13_SPINSTENOSIS	Spinal stenosis
5399	0	0.000554336	0	0.016818635	0.982627028	MEG3	finngen_R10_M13_SPINSTENOSIS	Spinal stenosis
2733	0	1.92E-09	0	0.028802862	0.971197136	TCF19	finngen_R10_M13_SPINSTENOSIS	Spinal stenosis
5520	3.95E-55	0.0002404	1.27E-53	0.006727205	0.993032395	DLK1	finngen_R10_M13_SPINSTENOSIS	Spinal stenosis
5431	2.01E-32	0.067219274	6.49E-33	0.020755399	0.912025327	IDH2	finngen_R10_M13_SPINSTENOSIS	Spinal stenosis
5028	1.14E-44	0.028248962	9.01E-45	0.021311981	0.950439057	KDM6B	finngen_R10_M13_SPINSTENOSIS	Spinal stenosis
2482	8.93E-33	0.000557849	1.52E-30	0.093758169	0.905683982	GPX1	finngen_R10_M13_SPINSTENOSIS	Spinal stenosis
4574	4.42E-10	0.010154409	7.59E-10	0.016478601	0.973366989	TET2	finngen_R10_M13_SPINSTENOSIS	Spinal stenosis
3826	8.13E-05	0.003458638	0.000435598	0.017545623	0.97847881	PI4KB	finngen_R10_M13_SPINSTENOSIS	Spinal stenosis
4566	8.43E-175	0.035411484	1.05E-174	0.042991318	0.921597198	CCL4	GCST90044547	Spinal stenosis (PheCode 720)
5803	2.82E-12	0.043390567	1.99E-12	0.029692296	0.926917137	PLA2G2C	GCST90044547	Spinal stenosis (PheCode 720)
8379	1.27E-40	0.052916947	9.67E-41	0.039297864	0.907785189	SORCS2	GCST90044553	Degeneration of intervertebral disc (PheCode 722.6)

**Table 3:** Binding energies of drug target proteins and drug molecule docking in each group

Protein	ZINC_ID	drug_name	affinity(kcal/mol)
PAK4	ZINC43131420	Fostamatinib	-8.1
ESR1	ZINC18825330	Genistein	-8.4
ESR1	ZINC3918428	Lasofoxifene	-11.1
PI4KB	ZINC43131420	Fostamatinib	-9.0

## Data Availability

The datasets used and analysed during the current study are available from the corresponding author on reasonable request.

## Abbreviations

ERα: Estrogen receptor alpha
HLA: Human leukocyte antigen
MR: Mendelian Randomization
PAK4: P21-activated kinase 4
PCs: Principal components
PI4KB: Phosphatidylinositol 4-kinase beta
PP: Posterior probabilities
TSS: Transcription start site
