# Supplementary material for: Novel Insight into the Therapeutic Targets for Spinal Degenerative Diseases Gained by a Post-Genome-Wide Association Study
Source: Int J Med Sci. 2026 May 18;23(7):2209–24. doi: 10.7150/ijms.127489 (PMC13280751; doi:10.7150/ijms.127489)
Supplement: Supplementary file 1 — Supplementary figures and tables. [file ijmsv23p2209s1.zip › Fig. S2.pdf]

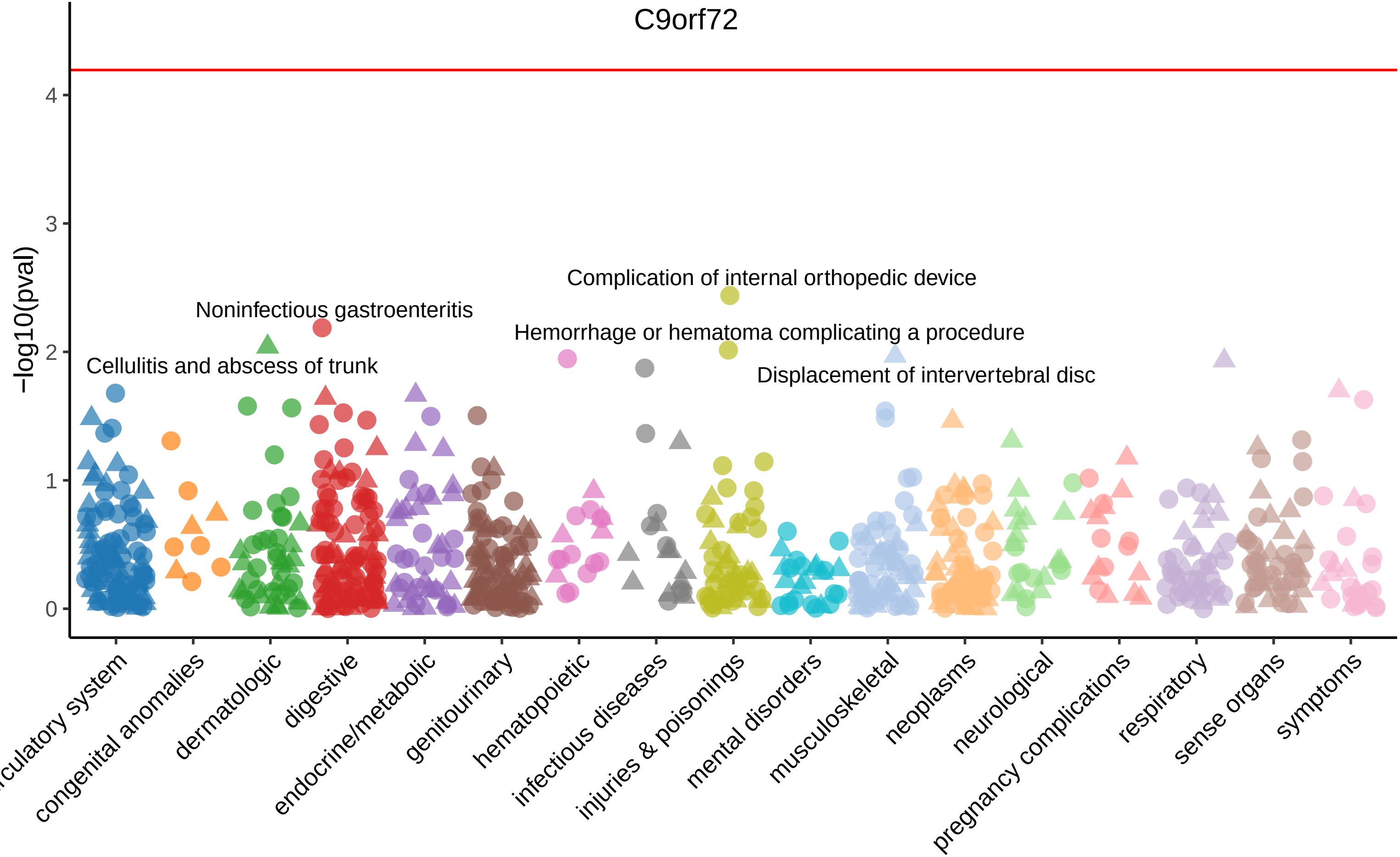
C9orf72
-log10(pval)
0
1
2
3
4
Complication of internal orthopedic device
Noninfectious gastroenteritis
Hemorrhage or hematoma complicating a procedure
Cellulitis and abscess of trunk
Displacement of intervertebral disc
circulatory system
congenital anomalies
dermatologic
digestive
endocrine/metabolic
genitourinary
hematopoietic
infectious diseases
injuries & poisonings
mental disorders
musculoskeletal
neoplasms
neurological
pregnancy complications
respiratory
sense organs
symptoms

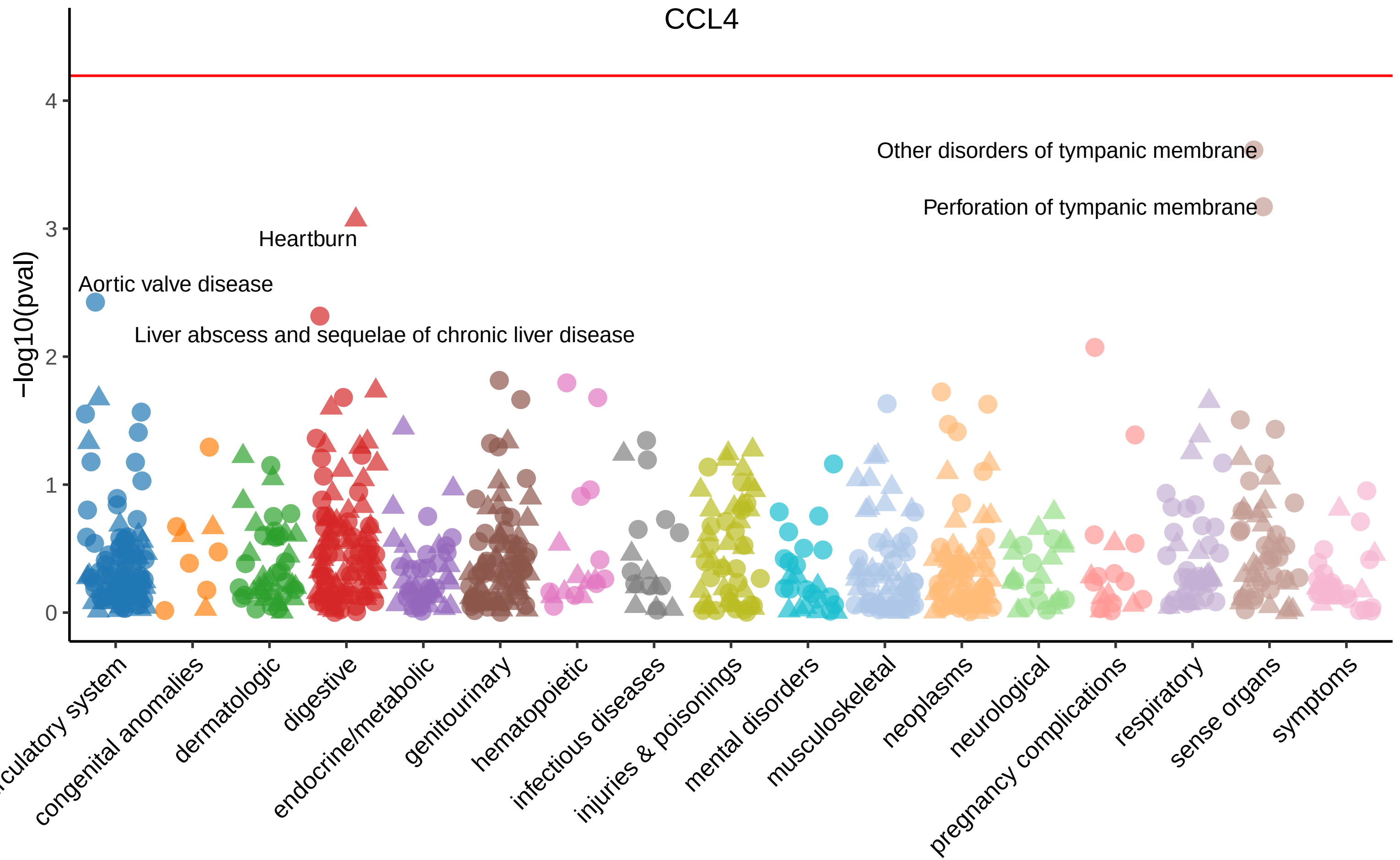
CCL4
-log10(pval)
4
3
2
1
0
Other disorders of tympanic membrane
Perforation of tympanic membrane
Heartburn
Aortic valve disease
Liver abscess and sequelae of chronic liver disease
circulatory system
congenital anomalies
dermatologic
digestive
endocrine/metabolic
genitourinary
hematopoietic
infectious diseases
injuries & poisonings
mental disorders
musculoskeletal
neoplasms
neurological
pregnancy complications
respiratory
sense organs
symptoms

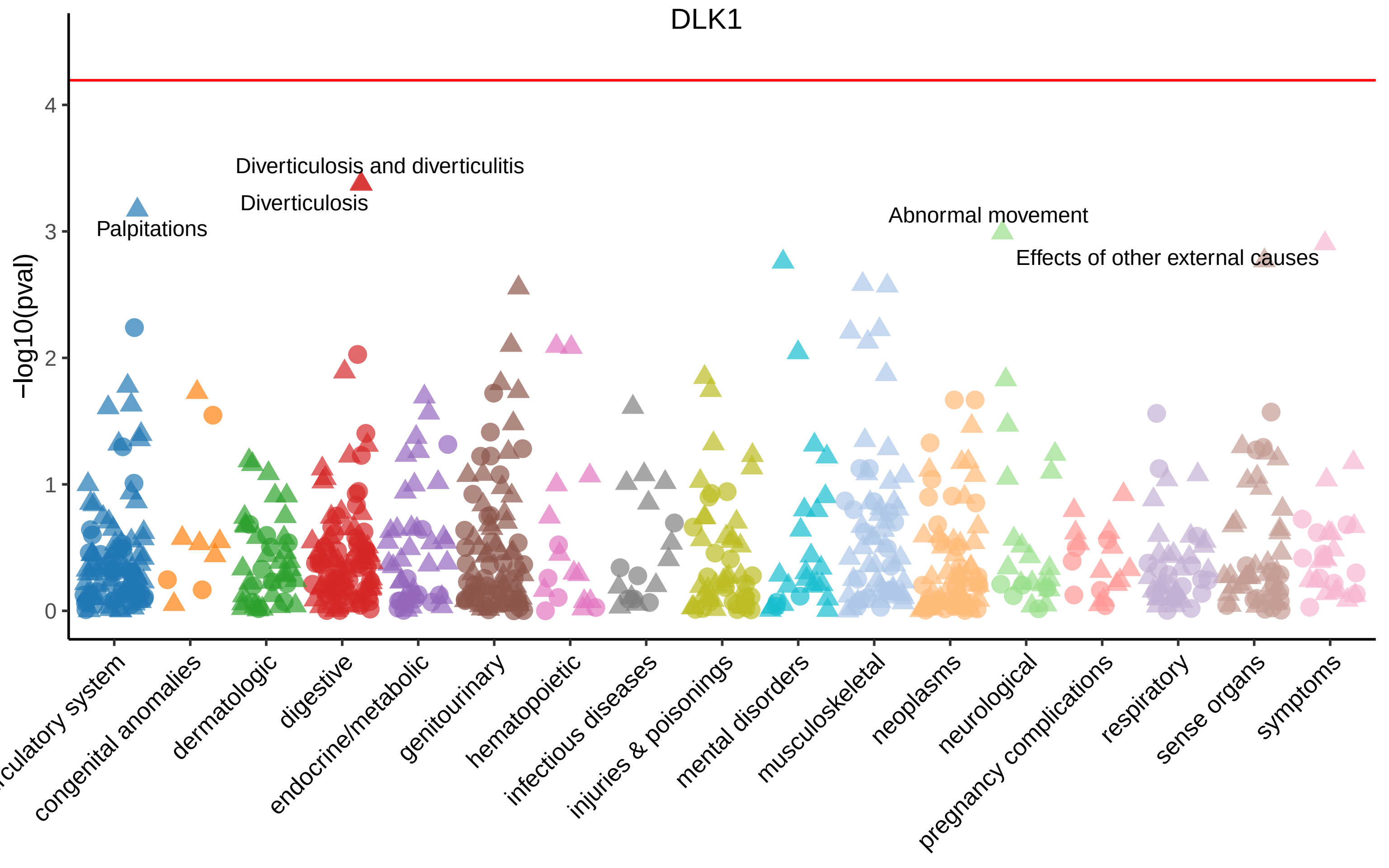
DLK1
-log10(pval)
0
1
2
3
4
Diverticulosis and diverticulitis
Diverticulosis
Palpitations
Abnormal movement
Effects of other external causes
circulatory system
congenital anomalies
dermatologic
digestive
endocrine/metabolic
genitourinary
hematopoietic
infectious diseases
injuries & poisonings
mental disorders
musculoskeletal
neoplasms
neurological
pregnancy complications
respiratory
sense organs
symptoms

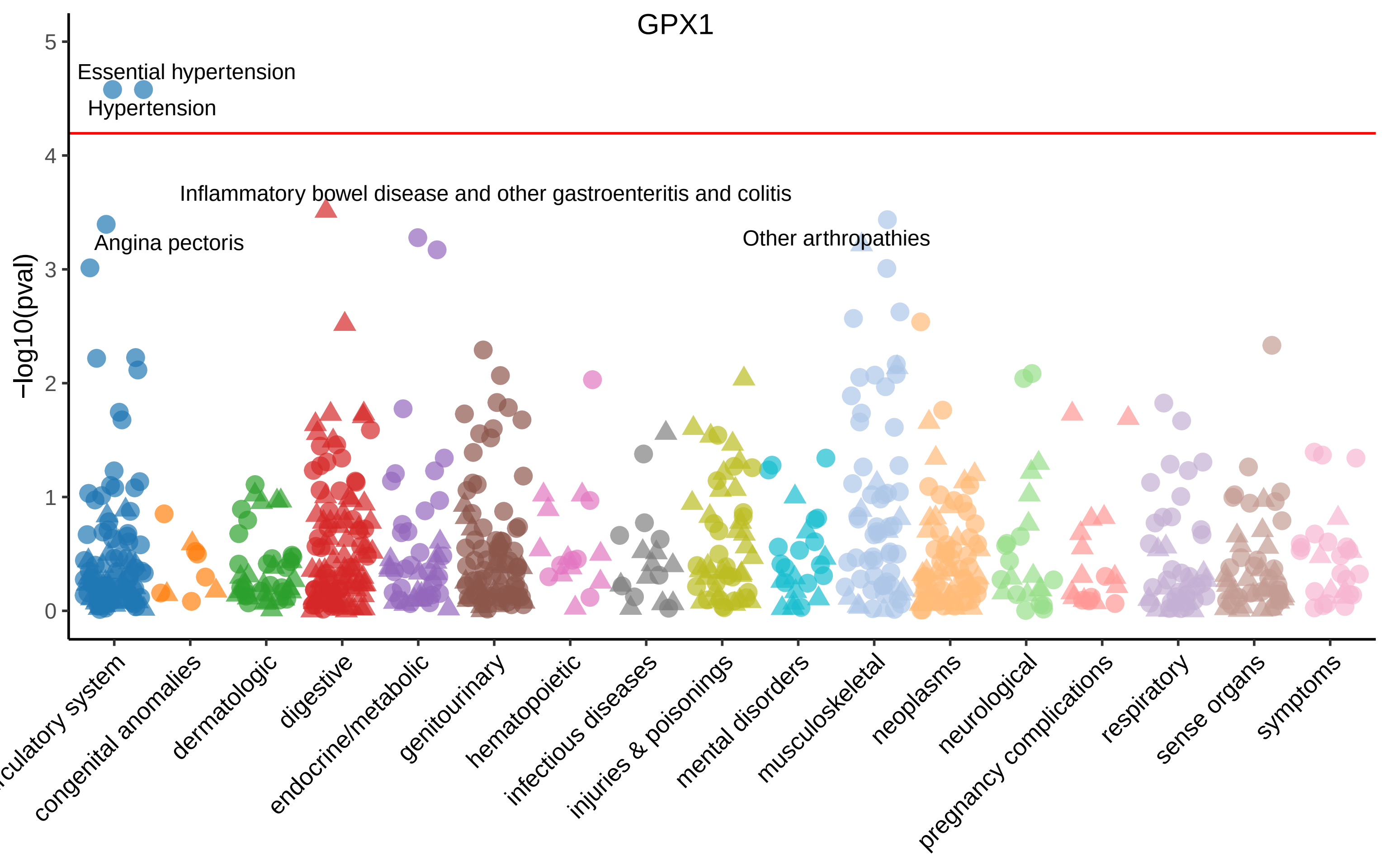
GPX1
Essential hypertension
Hypertension
Inflammatory bowel disease and other gastroenteritis and colitis
Angina pectoris
Other arthropathies
−log10(pval)
0
1
2
3
4
5
circulatory system
congenital anomalies
dermatologic
digestive
endocrine/metabolic
genitourinary
hematopoietic
infectious diseases
injuries & poisonings
mental disorders
musculoskeletal
neoplasms
neurological
pregnancy complications
respiratory
sense organs
symptoms

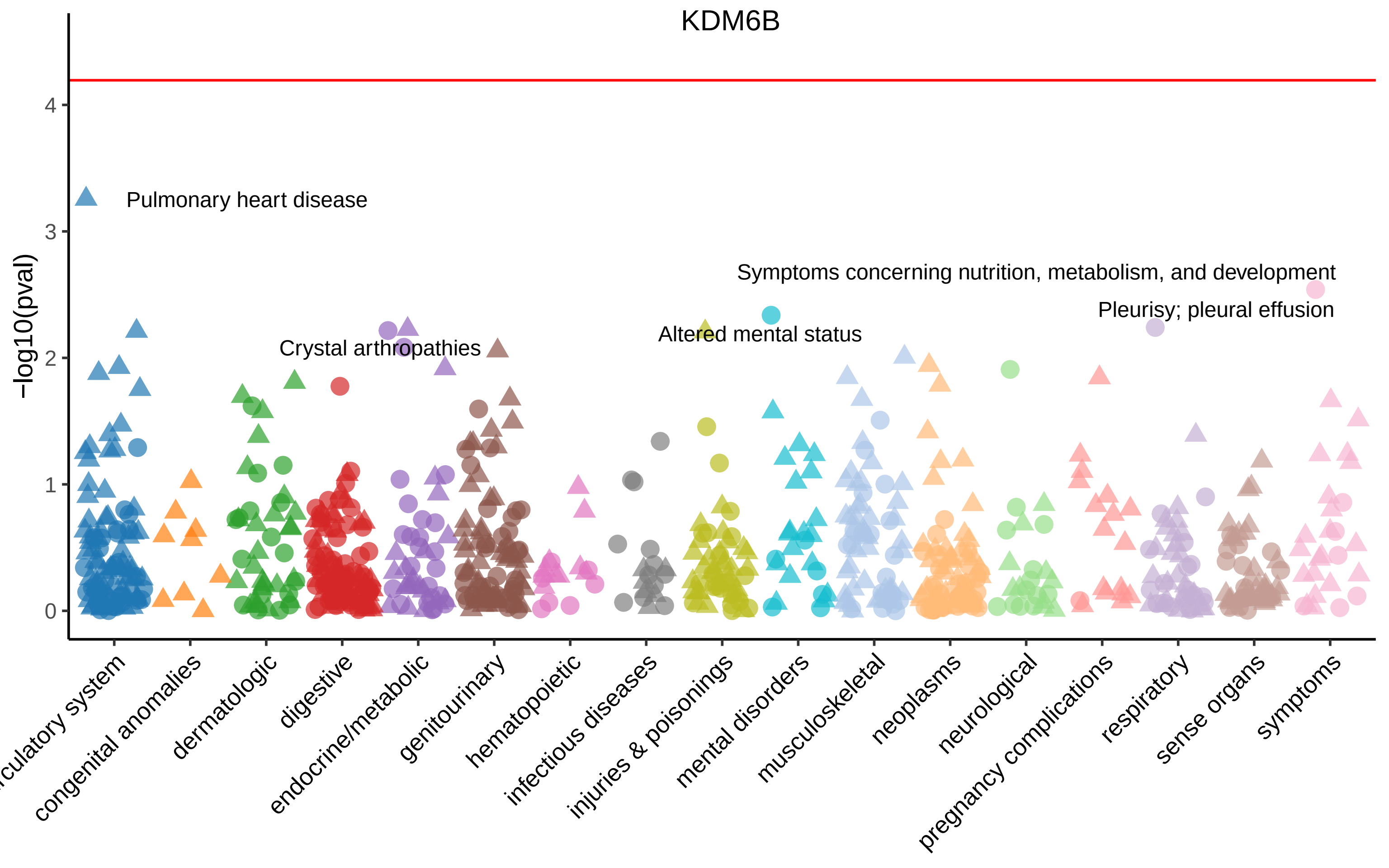
KDM6B
-log10(pval)
0
1
2
3
4
Pulmonary heart disease
Symptoms concerning nutrition, metabolism, and development
Pleurisy; pleural effusion
Altered mental status
Crystal arthropathies
culatory system
congenital anomalies
dermatologic
digestive
endocrine/metabolic
genitourinary
hematopoietic
infectious diseases
injuries & poisonings
mental disorders
musculoskeletal
neoplasms
neurological
pregnancy complications
respiratory
sense organs
symptoms

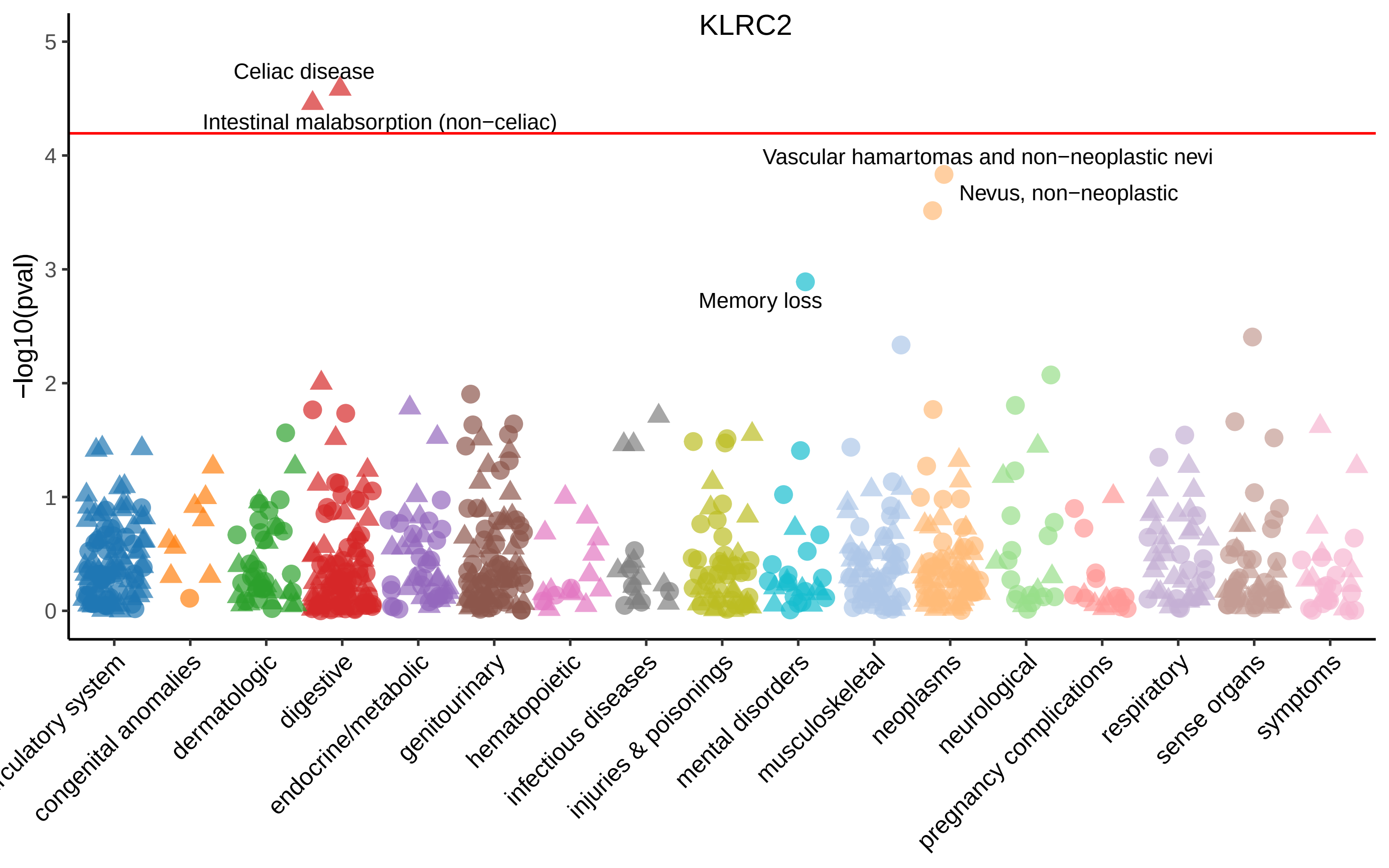

KLRC2
Celiac disease
Intestinal malabsorption (non−celiac)
Vascular hamartomas and non−neoplastic nevi
Nevus, non−neoplastic
Memory loss
−log10(pval)
0
1
2
3
4
5
circulatory system
congenital anomalies
dermatologic
digestive
endocrine/metabolic
genitourinary
hematopoietic
infectious diseases
injuries & poisonings
mental disorders
musculoskeletal
neoplasms
neurological
pregnancy complications
respiratory
sense organs
symptoms

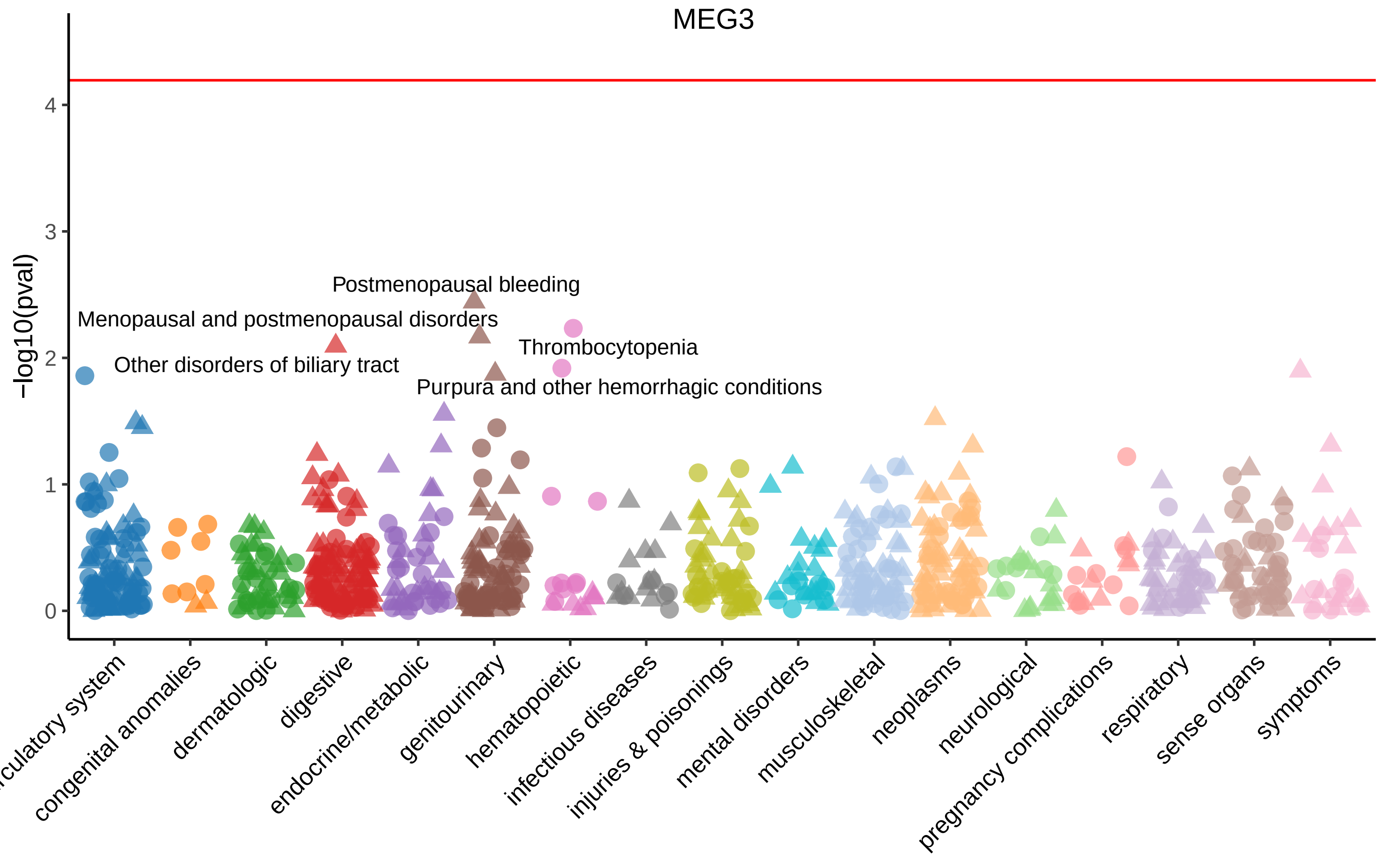
MEG3
−log10(pval)
4
3
2
1
0
Postmenopausal bleeding
Menopausal and postmenopausal disorders
Thrombocytopenia
Other disorders of biliary tract
Purpura and other hemorrhagic conditions
circulatory system
congenital anomalies
dermatologic
digestive
endocrine/metabolic
genitourinary
hematopoietic
infectious diseases
injuries & poisonings
mental disorders
musculoskeletal
neoplasms
neurological
pregnancy complications
respiratory
sense organs
symptoms

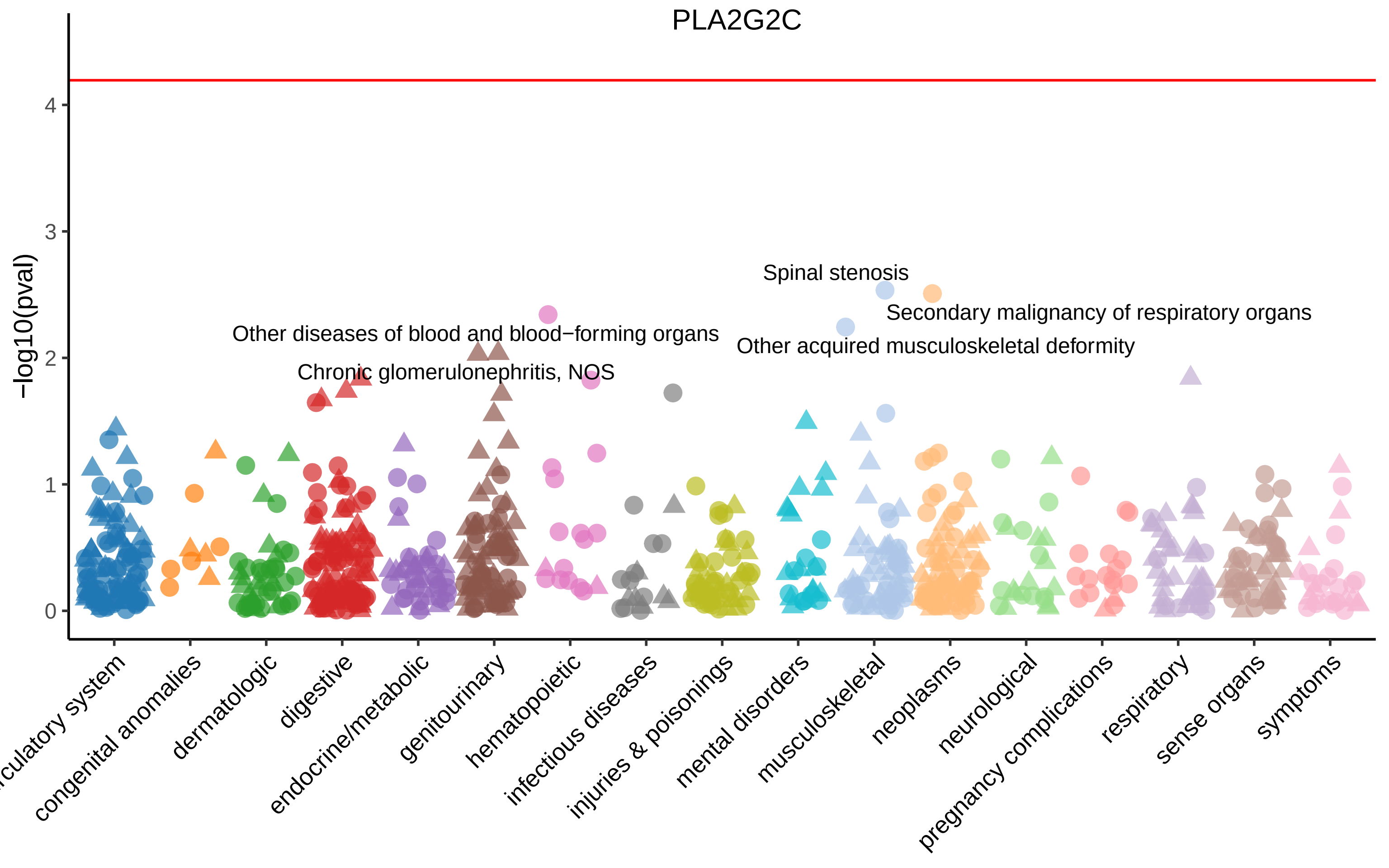
PLA2G2C
−log10(pval)
0
1
2
3
4
Spinal stenosis
Secondary malignancy of respiratory organs
Other diseases of blood and blood−forming organs
Other acquired musculoskeletal deformity
Chronic glomerulonephritis, NOS
circulatory system
congenital anomalies
dermatologic
digestive
endocrine/metabolic
genitourinary
hematopoietic
infectious diseases
injuries & poisonings
mental disorders
musculoskeletal
neoplasms
neurological
pregnancy complications
respiratory
sense organs
symptoms

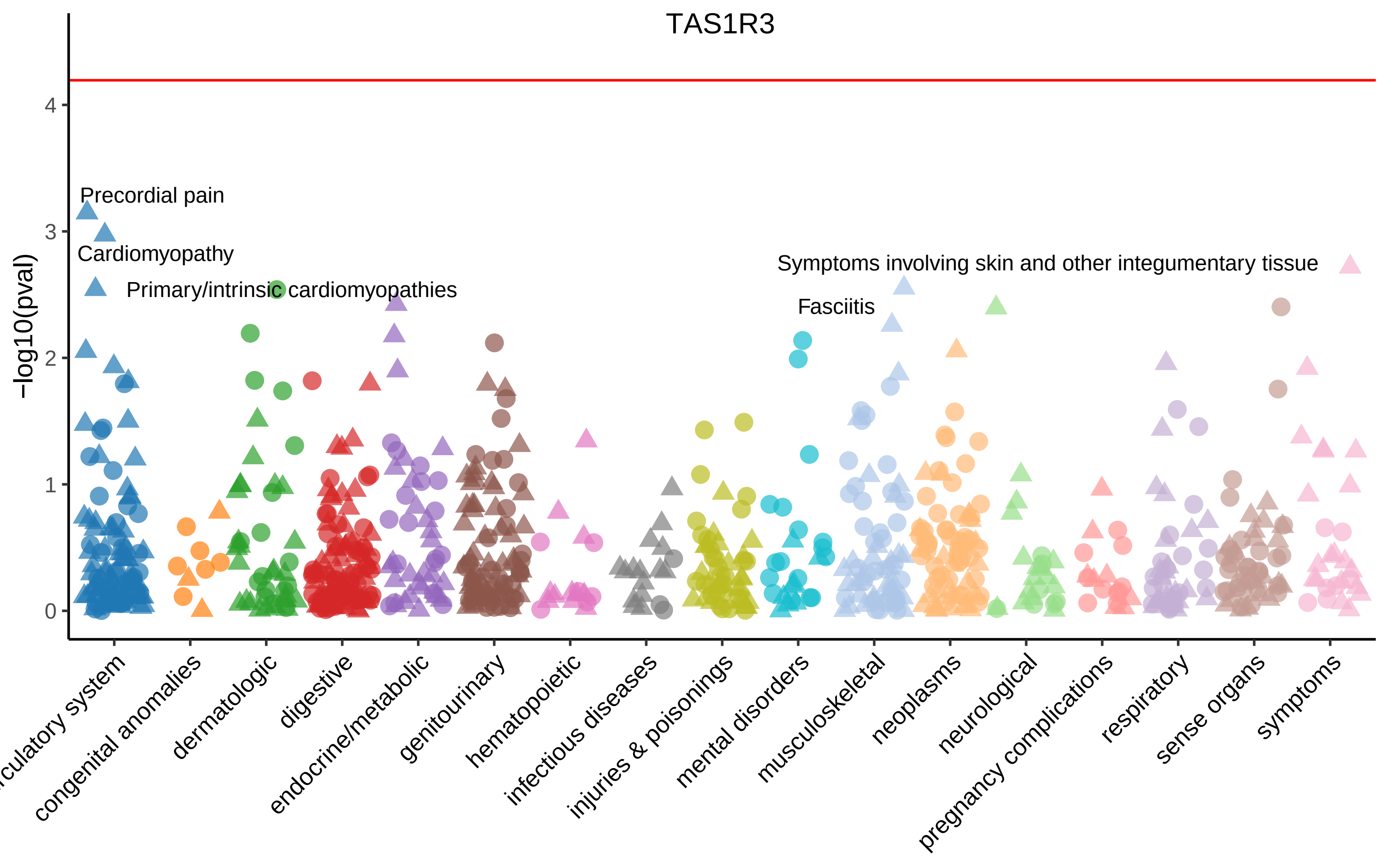

TAS1R3
−log10(pval)
0
1
2
3
4
Precordial pain
Cardiomyopathy
Primary/intrinsic cardiomyopathies
Symptoms involving skin and other integumentary tissue
Fasciitis
culatory system
congenital anomalies
dermatologic
digestive
endocrine/metabolic
genitourinary
hematopoietic
infectious diseases
injuries & poisonings
mental disorders
musculoskeletal
neoplasms
neurological
pregnancy complications
respiratory
sense organs
symptoms

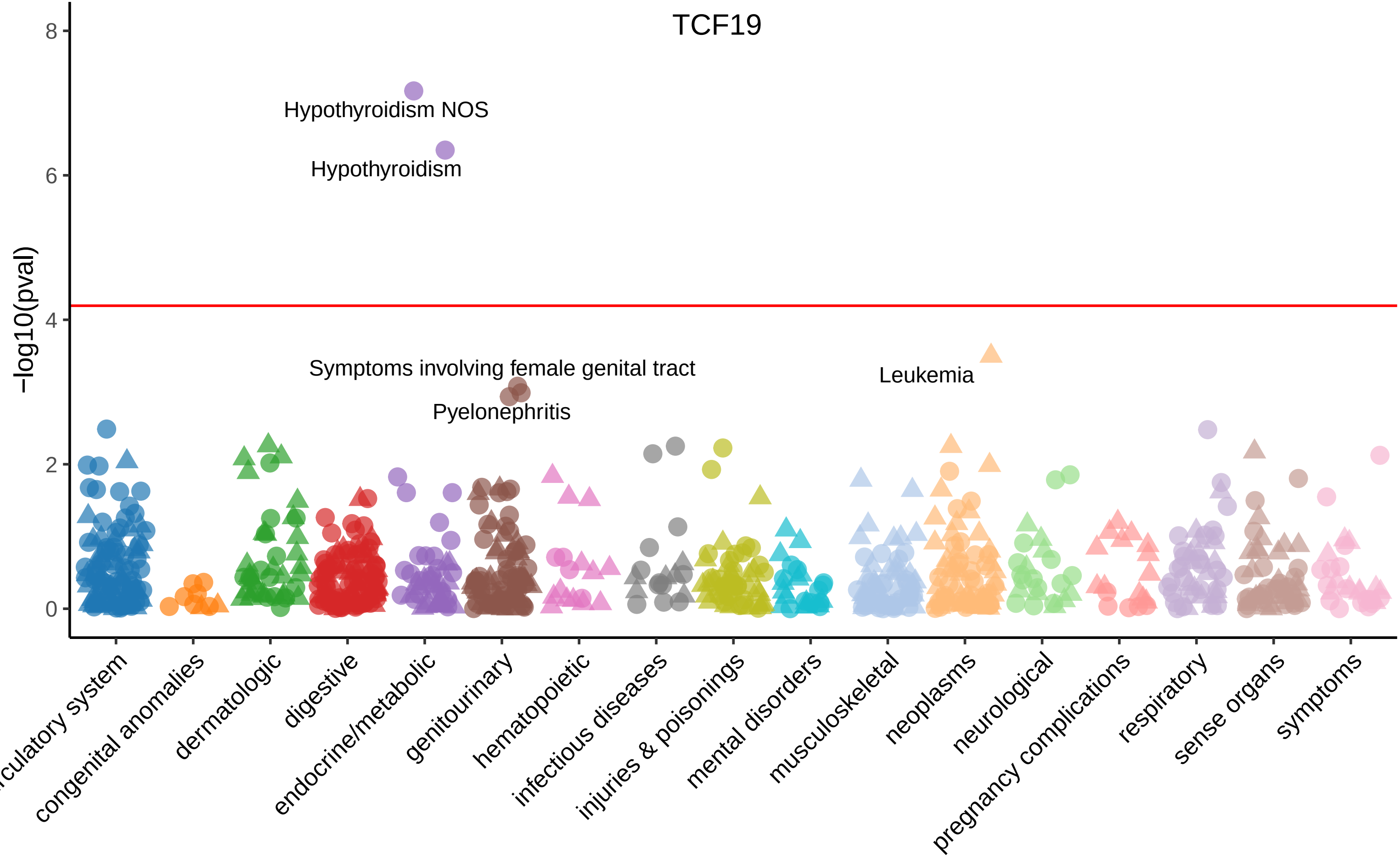
TCF19
-log10(pval)
8
6
4
2
0
Hypothyroidism NOS
Hypothyroidism
Symptoms involving female genital tract
Pyelonephritis
Leukemia
congenital anomalies
dermatologic
digestive
endocrine/metabolic
genitourinary
hematopoietic
infectious diseases
injuries & poisonings
mental disorders
musculoskeletal
neoplasms
neurological
pregnancy complications
respiratory
sense organs
symptoms

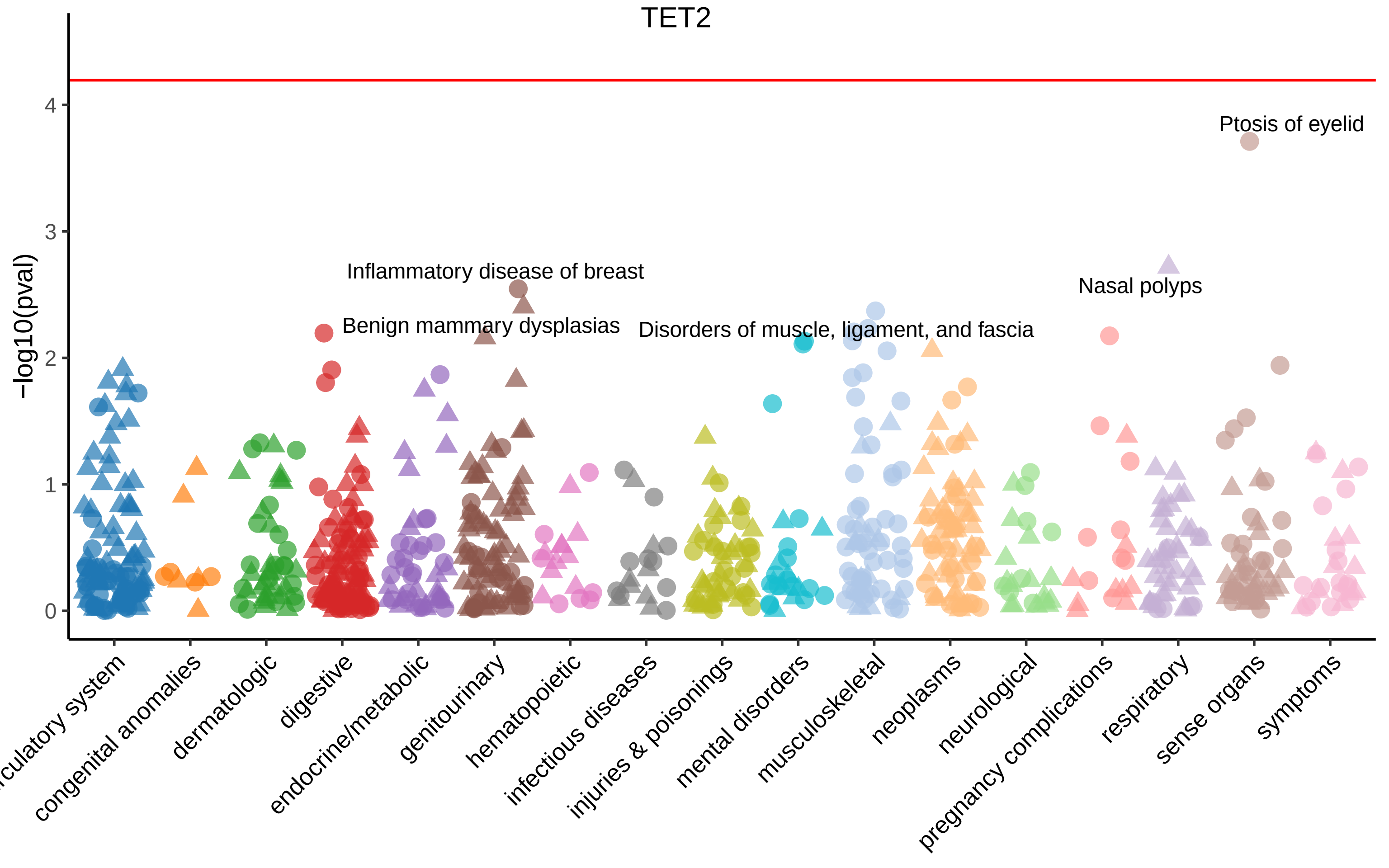
TET2
−log10(pval)
0
1
2
3
4
Ptosis of eyelid
Inflammatory disease of breast
Nasal polyps
Benign mammary dysplasias
Disorders of muscle, ligament, and fascia
rculatory system
congenital anomalies
dermatologic
digestive
endocrine/metabolic
genitourinary
hematopoietic
infectious diseases
injuries & poisonings
mental disorders
musculoskeletal
neoplasms
neurological
pregnancy complications
respiratory
sense organs
symptoms
