# Supplementary figures and images for: Novel Insight into the Therapeutic Targets for Spinal Degenerative Diseases Gained by a Post-Genome-Wide Association Study
Source: Int J Med Sci. 2026 May 18;23(7):2209–24. doi: 10.7150/ijms.127489 (PMC13280751; doi:10.7150/ijms.127489)

# forestplot\_GCST90080488

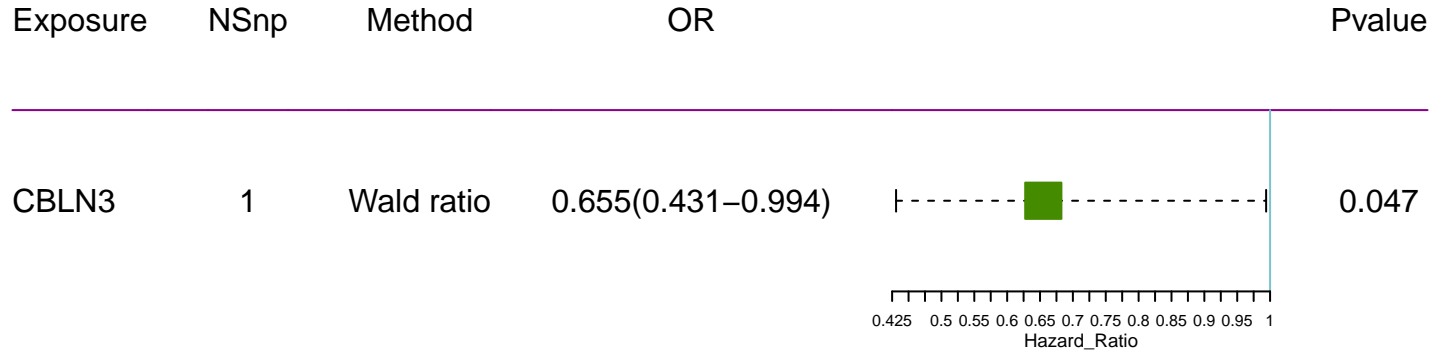

Supplement: Supplementary file 1 — Supplementary figures and tables. [file ijmsv23p2209s1.zip › Fig. S1.pdf]
